# Signed Tropicalization of Polar Cones

**DOI:** 10.1007/s10957-025-02732-2

**Published:** 2025-07-10

**Authors:** Marianne Akian, Xavier Allamigeon, Stéphane Gaubert, Sergeĭ Sergeev

**Affiliations:** 1 Inria and CMAP, École polytechnique, IP Paris, CNRS, Paris, France; 2 University of Birmingham, Edgbaston, B15 2TT United Kingdom

**Keywords:** Signed tropicalization, Polar cone, Completely positive, Copositive, Symmetrized tropical semiring, 15A80, 49N15, 90C24, 14T90

## Abstract

We study the tropical analogue of the notion of polar of a cone, working over the semiring of tropical numbers with signs. We characterize the cones which arise as polars of sets of tropically nonnegative vectors by an invariance property with respect to a tropical analogue of Fourier–Motzkin elimination. We also relate tropical polars with images by the nonarchimedean valuation of classical polars over real closed nonarchimedean fields and show, in particular, that for semi-algebraic sets over such fields, the operation of taking the polar commutes with the operation of signed valuation (keeping track both of the nonarchimedean valuation and sign). We apply these results to characterize images by the signed valuation of classical cones of matrices, including the cones of positive semidefinite matrices, completely positive matrices, completely positive semidefinite matrices, and their polars, including the cone of co-positive matrices, showing that hierarchies of classical cones collapse under tropicalization. We finally discuss an application of these ideas to optimization with signed tropical numbers.

## Introduction

### Motivation and Context

The present paper is motivated by a wish to develop a useful and fully functional analogue of optimization theory over the tropical semifield, which is the set of real numbers with adjoined $-\infty$, equipped with the operations of “addition” being the maximum of two numbers and “multiplication” being the ordinary addition. Since any real number exceeds $-\infty$, all real numbers are “positive” in the tropical sense, which entails that there is no tropical analogue of subtraction.

Much of tropical linear algebra, tropical convexity and their applications were developed in a “subtraction free” setting, see in particular [19, 21, 23, 43, 59], and the monographs [18, 36] and [41]. However, the link between classical notions (in optimization and real geometry) and their tropical analogues can be better understood by working over an extension of tropical numbers that encodes a sign information. This approach was developed by M. Plus [50], where the symmetrized extension of the tropical semifield was introduced, leading to a tropical analogue of Cramer theorem: see, e.g., the monograph by Baccelli et al. [13] and the articles by Akian, Gaubert and Guterman [1, 3]. This extension involves signed tropical numbers. The same numbers arose in Viro’s patchworking method, allowing one to construct parametric families of real algebraic curves with a prescribed topology, see Itenberg and Viro [39]. In a nutshell, Viro’s method shows that such a parametric family admits a piecewise-linear “log-limit”, a tropical curve. Signed tropical numbers have been used to study the tropicalization of linear programs, in relation with open complexity issues in game theory and linear programming, see Allamigeon et al. [7] and the references therein. Recent results in the setting of hyperfields [34, 40, 46, 57], or of semiring systems [5], have shown a growing interest in signed tropicalizations. Recent works by Loho, Skomra and Végh deal with signed extensions of tropical convexity [44, 45].

In classical convexity theory and in optimization, the notion of polar cone is a fundamental one, at the heart of the duality theory. A polar cone represents the family of half-spaces containing a given set. In the tropical setting, such half-spaces are defined by linear inequalities whose coefficients are tropical numbers with signs. Hence, tropical polars can be thought of as sets of vectors whose components are signed tropical numbers. This raises the question of characterizing the sets which arise as tropical polars, as well as computing the polars of the tropical analogues of fundamental classes of classical convex cones – including positive semidefinite matrices, completely positive and completely positive semidefinite matrices. We are also interested in relating tropical polar cones with non-archimedean valuations or “log-limits” of classical polar cones defined over a real closed non-archimedean field. We address these questions in the present paper.

### Main Results

After recalling some of the basic definitions, we focus on the polar cones of sets of tropically positive vectors (see Section 3). Our first main result, Theorem 3.3, characterizes these polar cones, showing they are precisely the sets of signed vectors that are closed in the topology of signed tropical numbers, and that are stable under a special operation of addition, encoding a Fourier–Motzkin elimination rule. We also show that polar cones are precisely the signed parts of the closed monotone precongruences initially studied by Gaubert and Katz [29]. Then, we study the relation between tropical polar cones and classical polar cones over ordered nonarchimedean fields. We consider the notion of *signed valuation*, taking into account not only the classical valuation but also the sign, already studied by Allamigeon, Gaubert and Skomra [12] as well as by Jell, Scheiderer and Yu [40]. Our second main result, Theorem 3.4, shows that for semialgebraic subsets over real closed non-archimedean fields with a surjective valuation to the additive group of real numbers, the operations of taking the polar and taking the signed valuation commute.

We next focus on the tropical analogues of classical cones (see Section 4). Here we first observe that the definitions of positive semidefinite matrices and completely positive matrices naturally extend to matrices over tropical signed numbers and we also define a tropical analogue of completely positive semidefinite matrices (see, e.g., Burgdorf, Laurent and Piovesan [17] for background on completely positive semidefinite matrices). Over the field of real numbers, and more generally, over any real closed field, the classical matrix cones form a hierarchy, with inclusions shown in Equation (3) below. Their polars, which include the cone of copositive matrices, yield a dual hierarchy displayed in Equation (4). In Theorem 4.4 and Theorem 4.6 we show that these hierarchies collapse under the tropicalization map, meaning that all inclusions in Equation (3) and Equation (4) become equalities in the tropical setting, or that the different cones in this hierarchy, over a real closed non-archimedean field, have the same images by the signed non-archimedean valuation. These results rely on surprisingly simple characterizations of tropical positive semidefinite matrices and tropical copositive matrices (see Theorem 4.1 and Theorem 4.5 below, extending a result of Yu [58]). We also use a characterization of Cartwright and Chan of tropical completely positive matrices [20] and rely on Theorem 3.4 (commutation between the operations of taking the signed valuation and the polar). In Section 5 we present some first elements of optimization over signed tropical numbers: the notion of minimization turns out to be more delicate than in the classical setting, owing to the restricted transivity of the “order” relation. As a simple but prominent example, we give an explicit solution of the minimization problem for univariate polynomial in Proposition 5.1. Then, we consider in Theorem 5.1 the tropical analogue of quadratic programming. We show that testing the nonnegativity of a quadratic form on the orthant is polynomial time solvable in the tropical world (this is perhaps surprising since the analogous problem in the classical world is co-NP complete). However, the general tropical quadratic optimization problem is NP-hard (Theorem 5.1).

The new contributions of this paper can be thus summarized as follows: 1) tropical polar cones are introduced as sets of signed vectors and characterized as closed sets stable under a special addition operation that eliminates some coordinates; 2) tropical analogues of classical matrix cones are introduced and characterized, it is shown in particular that the classical hierarchies of matrix cones and their polars collapse under the signed nonarchimedean valuation; 3) first elements of tropical optimization over signed tropical numbers, linking in particular the previous results with tropical quadratic optimization.

### Related Work

Tropical convexity, not taking signs into account, has been studied in great detail since the works of Zimmermann [59], see for example [9, 19, 22, 23, 41, 51] (among many other publications). Tropical convex sets and cones have a lot in common with the classical convex cones and convex sets: internal representation in terms of extremal vertices, external representation as intersection of halfspaces, separation theorems, etc.

Duality is one of the topics in tropical convexity that requires a quite different approach except in some special cases (see Gavalec and Zimmermann [32]), and the main reason for this is the absence of a genuine subtraction in tropical algebra. To this end, Gaubert and Katz [29] introduced and characterized *bi-polars* of tropical convex sets, avoiding the use of signed tropical numbers. In this setting, bi-polars were characterized as “closed pre-congruences”. Theorem 3.2 and Theorem 3.3 refine this result, showing that a closed pre-congruence can be canonically represented by a more concise object, the signed part of this pre-congruence, called “signed elimination cone”, which is characterized by a stability property with respect to Fourier–Motzkin type elimination.

Our results should be compared with recent ones by Loho and Végh [45], and then by Loho and Skomra [44], studying different types of convexity over symmetrized semirings (namely, TO-convexity, TC-convexity and hyperfield convexity). In particular, we are characterizing the tropical polars as sets that are closed and stable under a special addition operation that eliminates some coordinates, and this operation can be seen as closely related to the left-sum and TC-convexity defined in [44].

The study of tropicalizations of classical matrix cones goes back to the work of Yu [58], who characterized the image by the unsigned nonarchimedean valuation of the cone of positive semidefinite matrices. The present results refine this approach by taking the sign information into account. We rely on results of [12, 40] characterizing the images by the (signed) valuation of semialgebraic sets over ordered non-archimedean fields. The fact that nontrivial hierarchies of classical matrix cones collapse under the (signed) tropicalization map (Theorems 4.4 and 4.6) is reminiscent of the fact that the Helton-Nie conjecture, which was disproved by Scheiderer [53], has a valid tropical analogue as shown by Allamigeon, Gaubert and Skomra [11]; these different results reveal a loss of information inherent to the tropicalization process. We also note that the tropicalization of semialgebraic convex cones related to the moment problem was recently studied in Blekherman et al. [15].

## Basic Definitions

### The Symmetrized Tropical Semiring and its Relation with Real Puiseux Series

Let ${\mathbb {T}}$ be the *max-plus* or *tropical* semifield, that is the set of real numbers ${\mathbb {R}}$ with $-\infty$ endowed with $\max$ operation as addition, denoted $a\oplus b=\max (a,b)$ and usual addition as multiplication, denoted $a\odot b=a+b$, with zero $-\infty$, and unit 0. Recall that a *commutative semiring* is a set ${\mathcal {A}}$, equipped with an addition $(a,b)\mapsto a\oplus b$ that is associative, commutative, and has a neutral element ${\mathbb {0}}_{{\mathcal {A}}}$, together with a multiplication $(a,b)\mapsto a\odot b$ that is associative, commutative, has a unit $\mathbbm {1}_{{\mathcal {A}}}$, distributes over addition, and is such that ${\mathbb {0}}_{{\mathcal {A}}}$ is absorbing. It is a semifield if the nonzero elements have inverses for the multiplication. In ${\mathbb {T}}$, there are no opposites for the addition, which motivated the following construction of the symmetrized semiring by M. Plus [50]. The following definitions, except the order relation $\preccurlyeq$, can be found in the monograph by Baccelli et al. [13].

Consider the set ${\mathbb {T}}^2:={\mathbb {T}}\times {\mathbb {T}}$ endowed with the operations $\oplus$ and $\odot$ defined as:

$$\begin{aligned} (a^+,a^-) \oplus (b^+,b^-)&=(a^+\oplus b^+, a^- \oplus b^-),\\ (a^+,a^-) \odot (b^+,b^-)&= (a^+\odot b^+ \oplus a^- \odot b^-, a^+ \odot b^- \oplus a^- \odot b^+), \end{aligned}$$

with $(-\infty , -\infty )$ as the zero element and $(0, -\infty )$ as the unit element, also denoted by ${\mathbb {0}}_{{\mathbb {T}}^2}$ and $\mathbbm {1}_{{\mathbb {T}}^2}$, respectively. Then, ${\mathbb {T}}^2$ is a semiring. We define the operations $\ominus$ and $|\cdot |$

$$\begin{aligned} \ominus (a^+,a^-) = (a^-, a^+), \qquad |(a^+,a^-)| = a^+\oplus a^-\hspace{5.0pt}\!\!\!\!. \end{aligned}$$

Then, $a\in {\mathbb {T}}^2\mapsto \ominus a\in {\mathbb {T}}^2$ is an additive morphism, which allows us to write $a \oplus (\ominus b) = a \ominus b$ as usual. Moreover, $a\in {\mathbb {T}}^2\mapsto |a|\in {\mathbb {T}}$ is a semiring morphism.

The semiring ${\mathbb {T}}$ is endowed with a natural order, which coincides with the usual order on real numbers:

$$\begin{aligned} a\leqslant b\Leftrightarrow b=a\oplus b \Leftrightarrow b=a\oplus c\quad \text { for some }\; c. \end{aligned}$$

Based on this relation, the following relations can be defined on ${\mathbb {T}}^2$,

$$\begin{aligned} (a^+,a^-) \preccurlyeq (b^+,b^-)&\iff a^+ \oplus b^- \leqslant a^- \oplus b^+,\\ (a^+,a^-) \prec (b^+,b^-)&\iff a^+ \oplus b^- < a^- \oplus b^+, \\ (a^+,a^-) \nabla (b^+,b^-)&\iff a^+ \oplus b^- = a^- \oplus b^+. \end{aligned}$$

For $a,b\in {\mathbb {T}}^2$, we say that *a* *balances* *b* when $a \nabla b$. We define ${\mathbb {T}}^2_+ := \{(a^+,a^-)\in {\mathbb {T}}^2 \mid a^+>a^-\} \cup \{(-\infty ,-\infty )\}$, ${\mathbb {T}}^2_- := \{(a^+,a^-)\in {\mathbb {T}}^2 \mid a^+<a^-\}\cup \{(-\infty ,-\infty )\}$, ${\mathbb {T}}^2_{\pm }:= {\mathbb {T}}^2_+\cup {\mathbb {T}}^2_-$.

The two relations $\preccurlyeq$ and $\nabla$ are not transitive: for instance, if $a=(5,-\infty )$, $b=(7,7)$ and $c=(4,-\infty )$, we have $a\preccurlyeq b$, $a\nabla b$, $b\preccurlyeq c$, $b\nabla c$, but $a\preccurlyeq c$ and $a\nabla c$ do not hold. However, we have the following properties:

#### Proposition 2.1

Let $$a,b,c,d\in {\mathbb {T}}^2$$.

(i) $$a\preccurlyeq a$$ for any $$a\in {\mathbb {T}}^2$$;
(ii) $a\preccurlyeq b$ and $b\preccurlyeq a$ if and only if $a\nabla b$;
(iii) $a\preccurlyeq b$ if and only if $\ominus b\preccurlyeq \ominus a$;
(iv) $$a\preccurlyeq b \text { and }c\preccurlyeq d \implies a\oplus c\preccurlyeq b\oplus d$$;
(v) $$a\preccurlyeq b \text { and } c\succcurlyeq {\mathbb {0}}_{{\mathbb {T}}^2} \implies ac \preccurlyeq bc$$;
(vi) If $a\preccurlyeq b$ and $b\preccurlyeq c$ and $b\in {\mathbb {T}}^2_{\pm }$ then $a\preccurlyeq c$.

#### Proof

(i) to (v): Trivial.

(vi): As $a\preccurlyeq b$ and $b\preccurlyeq c$, we have $a^+\oplus b^-\leqslant a^-\oplus b^+$ and $b^+\oplus c^-\leqslant b^-\oplus c^+$. We consider the following three cases:

**Case 1:** $b^+> b^-$ (or equivalently $b\succ {\mathbb {0}}_{{\mathbb {T}}^2}$). In this case the inequalities $a\preccurlyeq b$ and $b\preccurlyeq c$ are equivalent to $a^+\leqslant a^-\oplus b^+$ and $b^+\oplus c^-\leqslant c^+$. Adding $c^-$ to the first of these inequalities, we obtain $a^+\oplus c^-\leqslant a^-\oplus b^+\oplus c^-$. Similarly, adding $a^-$ to the second inequality, we get $a^-\oplus b^+\oplus c^-\leqslant a^-\oplus c^+$. Using both resulting inequalities and the transitivity of $\leqslant$, we deduce $a^+\oplus c^-\leqslant a^-\oplus c^+$, which is $a\preccurlyeq c$.

**Case 2:** $b^-> b^+$. Then, $\ominus b \succ {\mathbb {0}}_{{\mathbb {T}}^2}$, and by (iii) $\ominus b \preccurlyeq \ominus a$ and $\ominus c\preccurlyeq \ominus b$. So using Case 1, we deduce that $\ominus c\preccurlyeq \ominus a$, which is equivalent to $a\preccurlyeq c$, by (iii).

**Case 3:** $$b^-=b^+=-\infty $$: trivial. $$\square $$

The order relation over ${\mathbb {T}}^2$ allows one to denote in a compact manner inequalities over the real numbers involving piecewise linear terms. For instance, $a\odot b\preccurlyeq c$ can be rewritten as follows with the usual notation:

$$\begin{aligned} \max (a^+ + b^+, a^- + b^-, c^-) \leqslant \max (a^-+b^+, a^++b^-, c^+). \end{aligned}$$

Observe that (vi) is a restricted transitivity property, requiring the intermediate term to belong to ${\mathbb {T}}^2_{\pm }$. In particular, the relations $\preccurlyeq$ and $\nabla$ restricted to ${\mathbb {T}}^2_{\pm }$ are transitive. Hence, there is a canonical refinement of the balance relation, that yields an equivalence relation ${\mathcal {R}}$ over ${\mathbb {T}}^2$:

$$\begin{aligned} (a^+,a^-) {\mathcal {R}} (b^+,b^-) \Leftrightarrow {\left\{ \begin{array}{ll} a^+ \oplus b^- = a^- \oplus b^+&  \;\text { if }\; (a^+, a^-), (b^+, b^-)\in {\mathbb {T}}^2_{\pm } ,\\ (a^+,a^-)=(b^+,b^-)&  \text { otherwise. } \end{array}\right. } \end{aligned}$$

One can check that ${\mathcal {R}}$ is compatible with the $\oplus$, $\odot$, and $\ominus$ operations, and with the relations $\preccurlyeq$, $\prec$, $\nabla$, which are therefore defined on the quotient ${\mathbb {T}}^2 / {\mathcal {R}}$.

#### Definition 2.1

The *symmetrized tropical semiring* is the quotient semiring $({\mathbb {S}}:={\mathbb {T}}^2 / {\mathcal {R}},\oplus ,\odot )$. We denote by ${\mathbb {0}}:=\overline{(-\infty ,-\infty )}$ the zero element and by $\mathbbm {1}:=\overline{(0, -\infty )}$ the unit element. We set ${\mathbb {S}}^+=\{x\in {\mathbb {S}}\mid x\succ {\mathbb {0}}\}\cup \{{\mathbb {0}}\}$, ${\mathbb {S}}^-=\{x\in {\mathbb {S}}\mid x\prec {\mathbb {0}}\}\cup \{{\mathbb {0}}\}$, ${\mathbb {S}}^\circ =\{x\in {\mathbb {S}}\mid x\nabla {\mathbb {0}}\}$, ${\mathbb {S}}^\vee = {\mathbb {S}}^+ \cup {\mathbb {S}}^-=\{{\bar{x}}\mid x\in {\mathbb {T}}^2_{\pm }\}$. We say that the elements of ${\mathbb {S}}^+,{\mathbb {S}}^-$ and ${\mathbb {S}}^\vee$ are *positive*, *negative*, and *signed*, respectively.

Note that here, we use the terms “positive” and “negative” in a weak sense, considering the zero element to be both positive and negative. Relating ${\mathbb {S}}^+$ and ${\mathbb {S}}^-$ with tropical pairs, it can be confirmed that ${\mathbb {S}}^+={\mathbb {T}}^2_+ / {\mathcal {R}}$ and ${\mathbb {S}}^-={\mathbb {T}}^2_-/{\mathcal {R}}$. Beware, however, that $\{x\in {\mathbb {S}}\mid x\succcurlyeq {\mathbb {0}}\}={\mathbb {S}}^+ \cup {\mathbb {S}}^\circ \ne {\mathbb {S}}^+$. Observe that any element $x\in {\mathbb {S}}^\vee$ can be written in a unique way as $x=x^+ \ominus x^-$ with $x^+,x^-\in {\mathbb {S}}^+$ and $x^+\odot x^-={\mathbb {0}}$. Moreover, the map $x\in {\mathbb {T}}\rightarrow \overline{(x,-\infty )}\in {\mathbb {S}}^+$ is an isomorphism of semirings and so we identify ${\mathbb {T}}$ with ${\mathbb {S}}^+$, and $-\infty$ with ${\mathbb {0}}$.

Note that $\preccurlyeq$ yields a total order on ${\mathbb {S}}^\vee$, and that ${\mathbb {S}}^\vee$ is unbounded in this order. We shall complete ${\mathbb {S}}^\vee$ by top and bottom elements, $\top$ and $\bot$, with the convention that $\ominus \top =\bot$, $\ominus \bot =\top$, and ${\mathbb {0}}\odot \top ={\mathbb {0}}\odot \bot ={\mathbb {0}}$.

Table 1 and Table 2 describe when $a\nabla b$ and $a\preccurlyeq b$ in terms of |*a*| and |*b*|. For example, we have $1\succ 0\succ -1\succ {\mathbb {0}}\succ \ominus -1\succ \ominus 0\succ \ominus 1.$

**Table 1:** Relation $$a\nabla b$$

$a\nabla b$	$b\in {\mathbb {S}}^+\setminus \{{\mathbb {0}}\}$	${\mathbb {S}}^-\setminus \{{\mathbb {0}}\}$	${\mathbb {S}}^{\circ }$
$a\in {\mathbb {S}}^+\setminus \{{\mathbb {0}}\}$	$|a|=|b|$	never	$|a|\leqslant |b|$
$a\in {\mathbb {S}}^-\setminus \{{\mathbb {0}}\}$	never	$|a|=|b|$	$|a|\leqslant |b|$
$a\in {\mathbb {S}}^{\circ }$	$|a|\geqslant |b|$	$|a|\geqslant |b|$	always

**Table 2:** Relation $$a\preccurlyeq b$$

$a \preccurlyeq b$	$b\in {\mathbb {S}}^+\setminus \{{\mathbb {0}}\}$	${\mathbb {S}}^-\setminus \{{\mathbb {0}}\}$	${\mathbb {S}}^{\circ }$
$a\in {\mathbb {S}}^+\setminus \{{\mathbb {0}}\}$	$|a|\leqslant |b|$	never	$|a|\leqslant |b|$
$a\in {\mathbb {S}}^-\setminus \{{\mathbb {0}}\}$	always	$|a|\geqslant |b|$	always
$a\in {\mathbb {S}}^{\circ }$	always	$|a|\geqslant |b|$	always

We now consider a field ${\mathcal {L}}$, equipped with a surjective nonarchimedean valuation map $\operatorname {val}$, that is a map from ${\mathcal {L}}$ to ${\mathbb {R}}\cup \{-\infty \}$, satisfying

$$\begin{aligned} \begin{aligned}&\operatorname {val}(x) = -\infty \iff x = 0, \\&\quad \forall x_{1}, x_{2} \in {\mathcal {L}}, \ \operatorname {val}(x_{1}x_{2}) = \operatorname {val}(x_{1}) + \operatorname {val}(x_{2}), \\&\quad \forall x_{1}, x_{2} \in {\mathcal {L}}, \ \operatorname {val}(x_{1} + x_{2}) \le \max (\operatorname {val}(x_{1}),\operatorname {val}(x_{2})). \end{aligned} \end{aligned}$$

Note that we use the “max-plus convention”, it is more frequent to call valuation the *opposite* of the map $\operatorname {val}$.

We also assume that ${\mathcal {L}}$ is equipped with a total order $\leqslant$, compatible with the operations of the field, so that ${\mathcal {L}}$ is an ordered field. We require also the nonarchimedean valuation $\operatorname {val}$ to be *convex*, meaning that the following property is satisfied:

$$\begin{aligned} x_{1} , \; x_{2} \in {\mathcal {L}}\, ,\;\text { and }\; 0 \le x_{2} \le x_{1} \implies \operatorname {val}(x_{2})\leqslant \operatorname {val}(x_{1}). \end{aligned}$$

An example of ordered, and in fact real closed, field with a convex valuation is provided by the field of real Hahn series ${\mathbb {R}}[[t^{{\mathbb {R}}}]]$, i.e., series with coefficients in ${\mathbb {R}}$, exponents in ${\mathbb {R}}$, such that the opposite of the support of the series is a well ordered set, see Engler and Prestel [26]. Then, the valuation of a series is its greatest exponent, and a series is positive if the coefficient of its monomial with greatest exponent is positive. One may also consider the subfield ${\mathbb {R}}\{\{t^{\mathbb {R}}\}\}$ of generalized Puiseux series with real coefficients. A non-zero element $f\in {\mathbb {R}}\{\{t^{\mathbb {R}}\}\}$ is a formal sum

$$\begin{aligned} f =\sum _{k\in {\mathbb {N}}} f_k t^{\lambda _k}, \end{aligned}$$

where the $f_k$ are real numbers, with $f_0\ne 0$, and $\lambda _k$ is a non-increasing sequence of real numbers converging to $-\infty$. We have $\operatorname {val}(f)=\lambda _0$. A series *f* is *positive* if $f_0>0$, and this defines a total order on ${\mathbb {R}}\{\{t^{\mathbb {R}}\}\}$. Markwig noted in [49] that it is a convenient choice of field to work out tropicalizations. It follows from [49] that this field is real closed. Another convenient choice of field is ${\mathbb {R}}\{\{t^{\mathbb {R}}\}\}_{\operatorname {cvg}}$, the subfield of ${\mathbb {R}}\{\{t^{\mathbb {R}}\}\}$ consisting of generalized Puiseux series that are absolutely convergent for $t>0$ large enough. This field is also real closed, see Van den Dries and Speisseger [55]. It has the advantage that the nonarchimedean valuation also has an analytic definition, as $\operatorname {val}(f)= \lim _{t\rightarrow \infty }\log (f(t))/\log (t)$. In the sequel, for simplicity, we will make a specific choice of field, taking ${\mathbb {K}}$ to be the field of formal generalized Puiseux series ${\mathbb {R}}\{\{t^{\mathbb {R}}\}\}$. The reader can verify that all the subsequent results carry over to any nonarchimedean real closed field with a surjective valuation to ${\mathbb {R}}$.

Following Allamigeon, Gaubert and Skomra [12], we define the *signed valuation* of an element $x\in {\mathcal {L}}$ to be the element $\operatorname {sval}(x)\in {\mathbb {S}}^\vee$ such that

$$\begin{aligned} \operatorname {sval}(x):={\left\{ \begin{array}{ll} \operatorname {val}(x) &  \textrm{if}\,\, x>0,\\ \ominus \operatorname {val}(x)&  \textrm{if}\,\, x<0,\\ {\mathbb {0}}&  \textrm{if}\,\, x=0, \end{array}\right. }\end{aligned}$$

where $\operatorname {val}(x)\in {\mathbb {T}}$ is identified as an element of ${\mathbb {S}}^+$. We observe that

$$\begin{aligned} x,y\in {\mathcal {L}}, x\leqslant y \implies \operatorname {sval}(x) \preccurlyeq \operatorname {sval}(y). \end{aligned}$$

As discussed in Akian, Gaubert and Rowen [5], ${\mathbb {S}}^\vee$ can be identified with the tropical real hyperfield of Viro [56] (also called real tropical hyperfield in Jell, Scheiderer and Yu [40] or the signed tropical hyperfield in Gunn [34]) and ${\mathbb {S}}$ is the hyperfield system of ${\mathbb {S}}^{\vee }$. Also recall that the real tropical hyperfield may be defined by modding out the field ${\mathbb {R}}\{\{t^{\mathbb {R}}\}\}$ by the multiplicative group consisting of positive absolutely convergent Puiseux series of valuation 0. The canonical order on ${\mathbb {R}}\{\{t^{\mathbb {R}}\}\}$ defines an order relation on this hyperfield, and the relation $\preccurlyeq$ extends this order to the signed tropical numbers. However, an inconvenience of the hyperfield is that the addition is multivalued, so it is more convenient to work here with the semiring ${\mathbb {S}}$, understanding that all previous statements can be translated to the setting of the hyperfields.

### Classical Matrix Cones

In this section, we recall the definition of several classical matrix cones. An $n\times m$ matrix *X* over an ordered field is said to be *nonnegative* if $X_{ij}\geqslant 0$ for all $i\in [n], \; j\in [m]$. We denote by $\operatorname {NN}_n$ the cone of nonnegative $n\times n$ matrices. A $n\times n$ matrix *X* is said to be *completely positive* of order *k* if there are nonnegative vectors $y^1,\dots ,y^n$ of size *k* such that

1 $$\begin{aligned} X_{ij}=\langle y^i, y^j\rangle , \qquad i,j\in [n], \end{aligned}$$

where $\langle y^i, y^j\rangle$ denotes the usual scalar product of vectors $y^i$ and $y^j$. Equivalently, $X=YY^T$ where *Y* is a $n\times k$ nonnegative matrix. We denote by $\operatorname {CP}_{n,k}$ the set of matrices of this form, and we denote by $\operatorname {CP}_n=\bigcup _{k\geqslant 1} \operatorname {CP}_{n,k}$ the set of *completely positive* matrices, which constitutes a convex cone. The above definitions make sense over any real closed field, in particular over ${\mathbb {R}}$ or ${\mathbb {K}}$. It will be convenient to make explicit the choice of the field, by writing for instance $\operatorname {CP}_n({\mathbb {K}})$ or $\operatorname {CP}_n({\mathbb {R}})$.

We denote by $\operatorname {PSD}_n$ the cone of positive semidefinite matrices of dimension $n\times n$. Following, e.g., Burgdorf, Laurent and Piovesan [17], we say that *X* is *completely positive semidefinite* of order *k* if there exist matrices ${Y}_1,\ldots ,{Y}_n\in \operatorname {PSD}_k$ such that

2 $$\begin{aligned} X_{ij}=\langle {Y}_i,{Y}_j\rangle , \qquad i,j\in [n], \end{aligned}$$

where $\langle Y,Z\rangle :=\operatorname {tr}(YZ^T)$ denotes the Frobenius scalar product. The set of matrices of this form is denoted by $\operatorname {CPSD}_{n,k}$, and by $\operatorname {CPSD}_n$ we denote the union of these sets, which constitutes a convex cone. The representation (1) corresponds to the special case of (2) in which all the matrices $Y_i$ are diagonal. It follows that $\operatorname {CP}_{n,k}\subset \operatorname {CPSD}_{n,k}$. The relation between the different classes of matrices considered so far is summarized in the following table:

3 $$\begin{aligned} \begin{array}{ccccc} \operatorname {CPSD}_{n,k}& \subset &  \operatorname {CPSD}_n&  \subset &  \operatorname {PSD}_n\cap \operatorname {NN}_n\\ \cup &  &  \cup \\ \operatorname {CP}_{n,k}&  \subset &  \operatorname {CP}_n. \end{array} \end{aligned}$$

Dually, denoting by $C^{\circ }:=\{x\mid \langle x, y\rangle \geqslant 0,\forall y\in C\}$ the polar of a set,

4 $$\begin{aligned} \begin{array}{ccccc} \operatorname {CPSD}_{n,k}^\circ &  \supset &  \operatorname {CPSD}_n^\circ &  \supset &  \operatorname {PSD}_n+ \operatorname {NN}_n\\ \cap & &  \cap \\ \operatorname {CP}_{n,k}^\circ & \supset &  \operatorname {CP}_n^{\circ }. \end{array} \end{aligned}$$

The inclusion $\operatorname {CPSD}_{n,k}\subset \operatorname {CPSD}_n$ is known to be strict for $k=2^{O(\sqrt{n})}$ (Prakash et al. [52]), whereas the inclusion $\operatorname {CP}_{n,k}\subset \operatorname {CP}_n$ is strict for $k=n^2/2 +O(n^{3/2})$ (Bomze, Schachinger and Ullrich [16]), see Remark 4.3 and Remark 4.4 for more information. It is known that $\operatorname {CP}_n\ne \operatorname {CPSD}_n\ne \operatorname {PSD}_n\cap \operatorname {NN}_n$ for $n=5$, see Fawzi et al. [27] for more information. Similarly, the dual hierarchy involving the polars of these cones is strict. Two of our main theorems imply that when taking the image by the valuation, and for $k\geqslant \max (n,\lfloor {n^2/4}\rfloor )$, each of these hierarchies collapses.

## Polars and Valuations

### Tropical Polars

The polar of a set over the field of Puiseux series is defined in a standard way.

#### Definition 3.1

Let $\varvec{A}$ be a subset of ${\mathbb {K}}^n$. Then the *polar cone* of this set is

$$\begin{aligned} \varvec{A}^{\circ }=\{\varvec{x}\in {\mathbb {K}}^n \mid \langle \varvec{x},\varvec{a}\rangle \geqslant \varvec{0} , \;\forall \varvec{a}\in \varvec{A}\}, \end{aligned}$$

where $\langle \varvec{x},\varvec{a}\rangle :=\varvec{\sum }_{i=1}^n \varvec{x}_i \varvec{a}_i$ is the usual scalar product.

In the tropical setting, we have a more delicate notion of polar introduced in [29].

#### Definition 3.2

([29]) The *two-sided polar* of a subset $A\subset {\mathbb {T}}^n$ is defined to be

$$\begin{aligned} A^\triangleright = \{(x^+,x^-) \in ({\mathbb {T}}^n)^2 \mid \langle x^+,a\rangle \geqslant \langle x^-,a\rangle , \;\forall a\in A\}. \end{aligned}$$

Dually, given a subset *B* of $({\mathbb {T}}^n)^2$, one considers the *one-sided polar*

$$\begin{aligned} B^\triangleleft = \{a \in {\mathbb {T}}^n \mid \langle x^+,a\rangle \geqslant \langle x^-,a\rangle , \;\forall (x^+,x^-)\in B\}. \end{aligned}$$

Here $\langle x,y\rangle =\bigoplus _{i=1}^n x_i\odot y_i$ is the usual tropical scalar product.

We equip ${\mathbb {T}}$ with the topology induced by the metric $d(x,y):= |\exp (x)-\exp (y)|$. We equip ${\mathbb {T}}^n$ and $({\mathbb {T}}^n)^2$ with the product topologies. We shall refer to these topologies as the *Euclidean* topologies. It is known that a closed tropical convex set is the intersection of the closed tropical half-spaces that contain it. It follows that for all $A\subset {\mathbb {T}}^n$, the “bipolar” $(A^\triangleright )^\triangleleft$ is the closed convex hull of *A*, see Cohen et al. [22]. The characterization of the “dual bipolar”, $(B^\triangleleft )^\triangleright$, requires the following notion.

#### Definition 3.3

A subset of $C\in ({\mathbb {T}}^n)^2$ is a *pre-congruence* if the following properties hold:

(i) If $(f^+,f^-)\in C$ then $(\lambda f^+,\lambda f^-)\in C$ for each $\lambda \in {\mathbb {T}}$;
(ii) For each $(f^+,f^-)\in C$ and $(g^+,g^-)\in C$ we have $(f^+\oplus g^+, f^- \oplus g^-)\in C$;
(iii) If $(f^+,f^-)\in C$, $(g^+,g^-)\in C$ and $f^-=g^+$, then $(f^+,g^-)\in C$.

We say that *C* is a *monotone pre-congruence* if, in addition, the following property holds

(iv) $(f^+,f^-)\in C$ if $f^+\geqslant f^-$ (where $\geqslant$ is the standard entrywise order).

In fact, the terminology *polar cone* is used in Gaubert and Katz [29] for the monotone pre-congruence notion, but since the term “polar” arises in different guises here, we change for a more explicit one. We also found it convenient here to make a change of the sign convention, so that, here, an element (*f*, *g*) encodes an inequality $f\geqslant g$, rather than the reverse.

#### Theorem 3.1

([29, Th. 10]) The two-sided polars of subsets of ${\mathbb {T}}^n$ are precisely the closed monotone pre-congruences of $({\mathbb {T}}^n)^2$. In particular, if *C* is a closed monotone pre-congruence, we have $C = (C^\triangleleft )^{\triangleright }$.

In what follows, we will define and characterize the signed polars of subsets of ${\mathbb {T}}^n$.

#### Definition 3.4

Let *A* be a subset of ${\mathbb {T}}^n$. Then the *signed polar* of this set is

5 $$\begin{aligned} A^{\circ }=\{x\in ({\mathbb {S}}^{\vee })^n\mid \langle x,a\rangle \succcurlyeq {\mathbb {0}},\;\forall a\in A), \end{aligned}$$

where $$\langle x,a\rangle = \langle x^+,a\rangle \ominus \langle x^-,a\rangle $$.

We recall that every element $x\in ({\mathbb {S}}^{\vee })^n$ can be written in a unique way as $x= x^+\ominus x^-$ where $x^+,x^-\in {\mathbb {T}}^n$ and $x^+_i\odot x^-_i={\mathbb {0}}$ for all $i\in [n]$ (remembering that ${\mathbb {T}}$ is identified with ${\mathbb {S}}^+$). Observe that, for all $x\in ({\mathbb {S}}^{\vee })^n$ and $a\in ({\mathbb {T}})^n$,

6 $$\begin{aligned} \langle x,a\rangle \succcurlyeq {\mathbb {0}}\Leftrightarrow \langle x^+,a\rangle \geqslant \langle x^-,a\rangle . \end{aligned}$$

We next establish an equivalence between signed polars and monotone pre-congruences, see Theorem 3.2 and Theorem 3.3.

#### Definition 3.5

A pair $(x^+,x^-)\in ({\mathbb {T}}^n)^2$ is called *signed* if for each *i* we have $x_i^+ \odot x^-_i={\mathbb {0}}$.

#### Definition 3.6

Given a subset $C\subset ({\mathbb {T}}^n)^2$, we denote by $C^{\vee }\subset C$ the *signed part* of *C* defined by

$$\begin{aligned} (x^+,x^-)\in C^{\vee }\Leftrightarrow (x^+,x^-)\in C\ \text { and }\ (x^+,x^-)\ \text { is signed. } \end{aligned}$$

#### Example 3.1

Consider the set $C\subset ({\mathbb {T}}^4)^2$ consisting of the following three vectors $x^1$, $x^2$, $x^3$:

$$\begin{aligned} x^1= \begin{pmatrix} (-\infty ,1)\\ (-\infty ,0)\\ (-3,-\infty )\\ (-\infty ,-5) \end{pmatrix},\quad x^2= \begin{pmatrix} (1,-\infty )\\ (0,-\infty )\\ (-\infty ,-3)\\ (-\infty , -5) \end{pmatrix},\quad x^3= \begin{pmatrix} (1,1)\\ (-\infty ,0)\\ (-3,-\infty )\\ (-5,-\infty ) \end{pmatrix}. \end{aligned}$$

Of these three vectors, $x^1$ and $x^2$ are signed and $x^3$ is not signed, so $C^{\vee }=\{x^1,x^2\}$.

Here and in the examples below we prefer to represent a vector in $({\mathbb {T}}^n)^2$ as a vector with *n* components in ${\mathbb {T}}^2$ rather than two vectors in ${\mathbb {T}}^n$. Obviously, these two representations are equivalent.

We will also use the following notation for the *diagonal* of $({\mathbb {T}}^n)^2$:

$$\begin{aligned} \varDelta ^n= \{(z,z)\mid z\in {\mathbb {T}}^n\}. \end{aligned}$$

We first show that every closed monotone pre-congruence of $({\mathbb {T}}^n)^2$ is determined by its signed part. To this end, we introduce the following notation. For all $(f^+,f^-)\in C$, we define $f^\vee =(f^{\vee +},f^{\vee -})$ by the following rule:

$$\begin{aligned} f^{\vee +}_i= {\left\{ \begin{array}{ll} f^+_i, &  \text { if } f^+_i\geqslant f^-_i,\\ {\mathbb {0}}, &  \text { if } f^+_i<f^-_i, \end{array}\right. }\quad f^{\vee -}_i= {\left\{ \begin{array}{ll} f^-_i, &  \text { if } f^-_i> f^+_i,\\ {\mathbb {0}}, &  \text { if } f^-_i\leqslant f^+_i. \end{array}\right. } \end{aligned}$$

We have the following property.

#### Lemma 3.1

Let $f\in ({\mathbb {T}}^n)^2$ and $x\in {\mathbb {T}}^n$. Then,

$$\begin{aligned} \langle f^+,x\rangle \geqslant \langle f^-,x\rangle \Leftrightarrow \langle f^{\vee +},x\rangle \geqslant \langle f^{\vee -},x\rangle . \end{aligned}$$

#### Proof

This follows from a general property of tropical half-spaces, which can be found for instance in [30, Section 3]. We reproduce the argument for the reader’s convenience. The relation $\langle f^+,x\rangle \geqslant \langle f^-,x\rangle$ can be rewritten as $\oplus _i f^+_i x_i \geqslant \oplus _i f^-_i x_i$. Let $J:=\{j \mid f^+_j\geqslant f^-_j\}$. Then, the previous inequality is equivalent to $\oplus _{j\in J} f^+_j x_j \geqslant \oplus _{i\not \in J} f^-_i x_i$ which is precisely $\langle f^{\vee +},x\rangle \geqslant \langle f^{\vee -},x\rangle$. $\square$

#### Proposition 3.1

Suppose that $C\subset ({\mathbb {T}}^n)^2$ is a closed monotone precongruence. Then,

(i) $$f\in C$$ implies $$f^\vee \in C$$;
(ii) $$C^\triangleleft = (C^{\vee })^\triangleleft $$;
(iii) $$C=C^{\vee }\oplus \varDelta ^n$$.

#### Proof

(i). As *C* is a closed monotone pre-congruence, by Theorem 3.1, we have $C=A^{\triangleright }$ for some subset $A\subset {\mathbb {T}}^n$. So $f= (f^+,f^-)\in C$ if and only if $\langle f^+,x\rangle \geqslant \langle f^-,x\rangle$ for all $x\in A$. Then, by Lemma 3.1, $f^\vee \in C$.
(ii). Since $C^{\vee } \subset C$, $C^\triangleleft \subset (C^{\vee })^\triangleleft$. If $a\in (C^{\vee })^\triangleleft$, then, for all $(f^+,f^-)\in C$, by (i), we have $(f^{\vee +},f^{\vee -})\in C^{\vee }$, and then we deduce from Lemma 3.1 that $\langle f^+,a\rangle \geqslant \langle f^-,a\rangle$, and so $a\in C^\triangleleft$.
(iii). We argue, as in the proof of (i), that $C=A^{\triangleright }$, so that $(f^+,f^-)\in C$ if and only if $\langle f^+,x\rangle \geqslant \langle f^-,x\rangle$ for all $x\in A$.

If $(f^+,f^-)\in C^{\vee }\oplus \varDelta ^n$ then $(f^+,f^-)=(g^+,g^-)\oplus (c,c)$ for some $(g^+,g^-)\in C^{\vee }$ and $c\in {\mathbb {T}}^n$ Obviously, $\langle f^+,x\rangle \geqslant \langle f^-,x\rangle$ for all $x\in A$ since $(g^+,g^-)$ and (*c*, *c*) also satisfy this property. Thus $C^{\vee }\oplus \varDelta ^n\subseteq C$.

Suppose now that $f\in C=A^{\triangleright }$. Then it follows from Lemma 3.1 that $f^\vee \in C$. Moreover, $f=f^{\vee }\oplus (c,c)$ where $c_i=\min (f^+_i,f^-_i)$ for all *i*, showing that $C\subset C^\vee \oplus \varDelta ^n$. $\square$

Let us introduce the following notation and operations:

#### Definition 3.7

For $z\in {\mathbb {T}}^n$ and $I\subseteq [n]$ we define vectors $z_I$ and $z_{\widehat{I}}$:

$$\begin{aligned} (z_I)_i= {\left\{ \begin{array}{ll} z_i, &  \text { for } i\in I,\\ {\mathbb {0}}&  \textrm{otherwise,} \end{array}\right. }\quad (z_{\widehat{I}})_i= {\left\{ \begin{array}{ll} z_i, &  \text { for } i\notin I,\\ {\mathbb {0}}&  \mathrm{otherwise.} \end{array}\right. } \end{aligned}$$

For $I=\{i\}$ we denote $z_{\widehat{i}}=z_{\widehat{\{i\}}}$.

#### Definition 3.8

Let $(x^+,x^-)\in ({\mathbb {T}}^n)^2$ and $(y^+,y^-)\in ({\mathbb {T}}^n)^2$ and suppose that $x^-_i=y^+_i$ for some $i\in [n]$. Then we define

$$\begin{aligned} (x^+,x^-)\oplus _i (y^+,y^-)=(x^+\oplus y^+_{\widehat{i}},\; x^-_{\widehat{i}}\oplus y^-). \end{aligned}$$

The next example shows how the transitivity of the order relation $\leqslant$ can be expressed in terms of the $\oplus _i$ operation.

#### Example 3.2

Suppose that a subset $A\subseteq {\mathbb {T}}^3$ is contained in the following signed halfspaces: $a_1\geqslant a_2$ and $a_2\geqslant a_3$. This means that the vectors $(0,\ominus 0,{\mathbb {0}})^T$ and $({\mathbb {0}}, 0,\ominus 0)^T$ are contained in $A^{\circ }$. The corresponding vectors $x^1$, $x^2$ with components in ${\mathbb {T}}^2$ and the vector $x^3=x^1\oplus _2 x^2$ are shown below:

$$\begin{aligned} x^1= \begin{pmatrix} (0,-\infty )\\ (-\infty ,0)\\ (-\infty ,-\infty ) \end{pmatrix},\quad x^2= \begin{pmatrix} (-\infty ,-\infty )\\ (0,-\infty )\\ (-\infty , 0) \end{pmatrix},\quad x^3= \begin{pmatrix} (0,-\infty )\\ (-\infty ,-\infty )\\ (-\infty , 0) \end{pmatrix}. \end{aligned}$$

The last vector corresponds to the vector $(0,{\mathbb {0}}, \ominus 0)^T$ and to the signed halfspace $a_1\geqslant a_3$, which also contains *A*. In this way, a transitivity property of the order relation $\leqslant$ is expressed in terms of stability under the operation $\oplus _2$.

#### Remark 3.1

The previous example shows that the new addition $\oplus _i$ can be interpreted as a tropical “Fourier–Motzkin elimination” of the variable $a_i$. Recall that Fourier–Motzkin elimination refers to the way to compute the projection $\pi (K)$ of a convex polyhedron *K* of ${\mathbb {R}}^n$, when for instance the projection $\pi$ consists in keeping the last $n-1$ entries, $\pi :(x_1,\ldots , x_n)\mapsto x'=(x_2,\ldots x_n)$. Indeed, the polyhedron *K* can always be rewritten as the set of $(x_1,x')\in {\mathbb {R}}^n$ such that $L_i(x')\leqslant x_1$, for all $i\in I$ and $x_1\leqslant R_j(x')$, for all $j\in J$ and $S_k(x')\leqslant 0$ for all $k\in K$, where *I*, *J*, *K* are finite sets and $L_i,R_j,S_k$ are affine functions. Then, Fourier–Motzkin elimination says that $\pi (K)$ is simply the set of $x'\in {\mathbb {R}}^{n-1}$ such that $S_k(x')\leqslant 0$ for all $k\in K$, and $L_i(x')\leqslant R_j(x')$ for all $(i,j)\in I\times J$, see [28] (see also [54]). That is we need only to eliminate $x_1$ in all pairs of inequalities involving $x_1$. See also Allamigeon et al. [8] for an algorithmic discussion of tropical Fourier–Motzkin elimination.

#### Remark 3.2

The addition $\oplus _i$ is also reminiscent of the bend relation (Giansiracusa and Giansiracusa [33], Maclagan and Rincón [47]), which plays a somehow similar role in the unsigned setting.

The next definition provides a natural extension of the $\oplus _i$ operation.

#### Definition 3.9

Let $(x^+,x^-)\in ({\mathbb {T}}^n)^2$ and $(y^+,y^-)\in ({\mathbb {T}}^n)^2$, and let $I=\{i\in [n]\mid x^-_i = y^+_i\}$. Then we define

$$\begin{aligned}&(x^+,x^-)\,{\widehat{\oplus }}\,(y^+,y^-)=(x^+\oplus y^+_{\widehat{I}},\; x^-_{\widehat{I}}\oplus y^-) \hspace{5.0pt},\\&\quad \text { and }\quad (x^+,x^-)\,{\widehat{\oplus }}^\vee \,(y^+,y^-)= \left( (x^+,x^-)\,{\widehat{\oplus }}\,(y^+,y^-)\right) ^\vee . \end{aligned}$$

In particular, if *I* is empty, $(x^+,x^-)\,{\widehat{\oplus }}\,(y^+,y^-) =(x^+,x^-){\oplus } (y^+,y^-)$.

#### Remark 3.3

If we identify $({\mathbb {T}}^n)^2$ to $({\mathbb {T}}^2)^n$ by $(x^+,x^-)\mapsto ((x_i^+,x_i^-))_{i=1,\ldots , n}$, we get that the operations $\,{\widehat{\oplus }}\,$ and $\,{\widehat{\oplus }}^\vee \,$ can be defined componentwise: $x\,{\widehat{\oplus }}\,y= ((x_i\,{\widehat{\oplus }}\,y_i))_{i=1,\ldots , n}$ for all $x=(x_i)_{i=1,\ldots , n}$, $y=(y_i)_{i=1,\ldots , n}$, with $x_i,y_i\in {\mathbb {T}}^2$, for all $i=1,\ldots , n$. The operation $\,{\widehat{\oplus }}^\vee \,$ can be defined equivalently as a binary operation on ${\mathbb {S}}^\vee$.

#### Example 3.3

Taking vectors $x^1$ and $x^2$ in Example 3.1 we see that

$$\begin{aligned} x^1= \begin{pmatrix} (-\infty ,1)\\ (-\infty ,0)\\ (-3,-\infty )\\ (-\infty ,-5) \end{pmatrix},\quad x^2= \begin{pmatrix} (1,-\infty )\\ (0,-\infty )\\ (-\infty ,-3)\\ (-\infty , -5) \end{pmatrix},\quad x^1\,{\widehat{\oplus }}^\vee \,x^2= \begin{pmatrix} (-\infty ,-\infty )\\ (-\infty ,-\infty )\\ (-3,-\infty )\\ (-\infty ,-5) \end{pmatrix}. \end{aligned}$$

#### Definition 3.10

A set $R\subset ({\mathbb {T}}^n)^2$ is called a *signed elimination cone* if each pair $(x,y)\in R$ is signed and the following properties hold:

(i) $$(x^+,{\mathbb {0}})\in R$$ for each $$x^+\in {\mathbb {T}}^n$$;
(ii) If $(x^+,x^-)\in R$ then $(\lambda x^+,\lambda x^-)\in R$ for each $\lambda \in {\mathbb {T}}$;
(iii) For each $(x^+,x^-)\in R$ and $(y^+,y^-)\in R$ we have $((x^+,x^-)\oplus (y^+,y^-))^{\vee }\in R$;
(iv) For each $(x^+,x^-)\in R$ and $(y^+,y^-)\in R$ and *i* such that $x^-_i=y^+_i$, we have $((x^+,x^-)\oplus _i (y^+,y^-))^{\vee }\in R$.

We will see below (Proposition 3.3) that the last two properties can be replaced by the stability of *R* under the $\,{\widehat{\oplus }}^\vee \,$ operation.

The terminology “signed elimination cone” refers to the analogy with Fourier-Motzkin elimination which was already discussed in Remark 3.1 and Example 3.2. Below we give yet another example where the stability under operation in Item (iv) for $i=1$ allows one to express the elimination of variable $a_1$ by a Fourier–Motzkin type approach.

#### Example 3.4

Consider the following four vectors whose components are tropical signed numbers: $(1\;, \ominus 1,\; 3,\; \ominus 2)^T$, $(2,\; \ominus 4,\; 1,\; \ominus 4)^T$, $(\ominus 3,\; 2,\; \ominus 4,\; 1)^T$ and $(\ominus 1,\; 3,\; 2,\; \ominus 3)^T$. They correspond to the following vectors with components in ${\mathbb {T}}^2$:

$$\begin{aligned} x^1= \begin{pmatrix} (1,-\infty )\\ (-\infty ,1)\\ (3,-\infty )\\ (-\infty ,2) \end{pmatrix},\ x^2= \begin{pmatrix} (2,-\infty )\\ (-\infty ,4)\\ (1,-\infty )\\ (-\infty ,4) \end{pmatrix},\quad x^3= \begin{pmatrix} (-\infty ,3)\\ (2,-\infty )\\ (-\infty ,4)\\ (1,-\infty ) \end{pmatrix}, x^4= \begin{pmatrix} (-\infty ,1)\\ (3,-\infty )\\ (2,-\infty )\\ (-\infty ,3) \end{pmatrix}. \end{aligned}$$

We have four ways to eliminate the first component by means of $\oplus _1$ operation: $y^1=((-2) x^3 \oplus _1 x^1)^{\vee }$, $y^2=(x^4\oplus _1 x^1)^{\vee }$, $y^3=((-1)x^3\oplus _1 x^2 )^{\vee }$ and $y^4=(x^4 \oplus _1 (-1) x^2)^{\vee }$ and it can be checked that the resulting vectors with components in ${\mathbb {T}}^2$ correspond to the following vectors with tropical signed components:

$$\begin{aligned} \begin{aligned} y^1&= \begin{pmatrix} (-\infty ,-\infty )\\ (-\infty , 1)\\ (3,-\infty )\\ (-\infty ,2) \end{pmatrix}\sim \begin{pmatrix} {\mathbb {0}}\\ \ominus 1\\ 3\\ \ominus 2 \end{pmatrix},\quad y^2= \begin{pmatrix} (-\infty ,-\infty )\\ (3, -\infty )\\ (3,-\infty )\\ (-\infty ,3) \end{pmatrix}\sim \begin{pmatrix} {\mathbb {0}}\\ 3\\ 3\\ \ominus 3 \end{pmatrix},\\ y^3&= \begin{pmatrix} (-\infty ,-\infty )\\ (-\infty , 4)\\ (-\infty ,3)\\ (-\infty ,4) \end{pmatrix}\sim \begin{pmatrix} {\mathbb {0}}\\ \ominus 4\\ \ominus 3\\ \ominus 4 \end{pmatrix},\quad y^4= \begin{pmatrix} (-\infty ,-\infty )\\ (3, -\infty )\\ (2,-\infty )\\ (-\infty ,3) \end{pmatrix}\sim \begin{pmatrix} {\mathbb {0}}\\ 3\\ 2\\ \ominus 3 \end{pmatrix}. \end{aligned} \end{aligned}$$

In particular, the signed vectors $(-2)x^3$ and $x^1$ encode the relations $a_2 \oplus (-1)a_4\geqslant 1 a_1 \oplus 2a_3$ and $1a_1 \oplus 3 a_3 \geqslant 1a_2 \oplus 2a_4$, respectively. By adding $3a_3$ to the former relation, we deduce that $a_2 \oplus (-1)a_4 \oplus 3a_3 \geqslant 1a_1 \oplus 3a_3 \geqslant 1a_2 \oplus 2a_4$, and after eliminating terms which cannot be active, we deduce that $3a_3 \geqslant 1a_2 \oplus 2a_4$, which is precisely the inequality expressed by the element $y^1=((-2)x^3 \oplus _1 x^1)^{\vee }$.

We shall identify $({\mathbb {T}}^n)^2$ to $({\mathbb {T}}^2)^n$. In this way a subset of $({\mathbb {T}}^n)^2$ consisting only of signed pairs can be identified with a subset of $({\mathbb {T}}^2_{\pm })^n$ or equivalently to a subset of $({\mathbb {S}}^{\vee })^n$, and vice versa. We shall denote by $\imath$ the canonical identification map from $({\mathbb {S}}^{\vee })^n$ to the set of signed pairs of $({\mathbb {T}}^n)^2$. In particular, the *signed polar* $U = A^\circ$ of a subset $A\subset {\mathbb {T}}^n$ which was defined as a subset of $({\mathbb {S}}^{\vee })^n$ gives rises to subset $\imath (U) \subset ({\mathbb {T}}^n)^2$ consisting of signed elements. We also recall that $({\mathbb {S}}^{\vee })^n$ is equipped with the product topology defined by considering the order topology on ${\mathbb {S}}^{\vee }$.

#### Theorem 3.2

Let $R\subset ({\mathbb {T}}^n)^2$. The following assertions are equivalent:

(i) *R* is a closed signed elimination cone.
(ii) There exists a closed monotone pre-congruence *C* of ${\mathbb {T}}^n$ such that *R* is the signed part of *C*.

#### Theorem 3.3

Let $U\subset ({\mathbb {S}}^{\vee })^n$. The following assertions are equivalent:

(i) The image $\imath (U)\subset ({\mathbb {T}}^n)^2$ by the canonical map is a signed elimination cone, and *U* is closed in $({\mathbb {S}}^{\vee })^n$.
(ii) There exists a subset $A\subset {\mathbb {T}}^n$ such that $U= A^\circ$.

The proof of Theorem 3.2 and of Theorem 3.3 relies on a series of auxiliary results.

#### Proposition 3.2

Let *C* be a closed monotone pre-congruence. Then $C^{\vee }$ is a signed elimination cone.

#### Proof

We need to check that $C^{\vee }$ satisfies the properties (i)-(iv) of Definition 3.10.

(i): if *C* is a monotone pre-congruence, then, by  Definition 3.3, (iv), for all $x^+\in {\mathbb {T}}^n$, $(x^+,{\mathbb {0}})\in C$, and since $(x^+,{\mathbb {0}})$ is signed, $(x^+,{\mathbb {0}})\in C^{\vee }$.

(ii): obvious from Definition 3.3 part (i).

(iii): suppose that $(x^+,x^-)\in C^\vee$ and $(y^+,y^-)\in C^\vee$. Then, by Definition 3.3, (ii), we have $(x^+,x^-)\oplus (y^+,y^-)\in C$. Then, by Proposition 3.1 part (i), $((x^+,x^-)\oplus (y^+,y^-))^\vee \in C^\vee$.

(iv): Let $(x^+,x^-)\in C^{\vee }$ and $(y^+,y^-)\in C^{\vee }$ with $x^-_i=y^+_i=t$. Then we have

$$\begin{aligned} x^-=x^-_{\widehat{i}}\oplus te_i,\quad y^+=y^+_{\widehat{i}}\oplus te_i. \end{aligned}$$

Our purpose is to show that $(x^+\oplus y^+_{\widehat{i}},x^-_{\widehat{i}}\oplus y^-)^{\vee }\in C^{\vee }$. We have $(x^+,x^-)\in C$ and by monotonicity of *C*, $(x^-,x^-_{\widehat{i}})\in C$, hence, by transitivity of *C*, $(x^+,x^-_{\widehat{i}})\in C$. Similarly, $(x^-,te_i)\in C$ by monotonicity, and so, $(x^+,t e_i)$ holds by transitivity.

As $(y^+_{\widehat{i}},y^+_{\widehat{i}})\in C$, we can add it to $(x^+,te_i)$ and then we obtain $(x^+\oplus y^+_{\widehat{i}}, y^+)\in C$. Using transitivity again, we obtain $(x^+\oplus y^+_{\widehat{i}}, y^-)\in C$.

As $(x^+,x^-_{\widehat{i}})\in C$, we can add it to $(x^+\oplus y^+_{\widehat{i}},y^-)$ and obtain $(x^+\oplus y^+_{\widehat{i}}, y^-\oplus x^-_{\widehat{i}}) \in C$. Using again Proposition 3.1 part (i), we deduce that $(x^+\oplus y^+_{\widehat{i}}, y^-\oplus x^-_{\widehat{i}})^\vee \in C^\vee$. $\square$

For all $z\in {\mathbb {T}}^n$, we denote by $\operatorname {supp}(z)=\{i\in [n]\mid z_i\ne {\mathbb {0}}\}$ the *support* of *z*. In what follows we will also consistently drop the $\odot$ sign.

#### Lemma 3.2

Let *R* be a signed elimination cone and take any $x=(x^+,x^-)\in R$ and suppose that $y=(y^+,y^-)$ is signed and that $y\succcurlyeq x$. Then $y\in R$.

We note that,

7 $$\begin{aligned} \text { for }x \text { and }y\text { signed, } y\succcurlyeq x \iff (y^+\geqslant x^+ \text { and } x^- \geqslant y^-). \end{aligned}$$

#### Proof

We first show that $(x^+,x^-_ie_i)\in R$ for all $i\in \operatorname {supp}(x^-)$. More generally, we will show that $(x^+,x^-_{\widehat{I}})\in R$ for all subsets $I\subset \operatorname {supp}(x^-)$. For this, it is enough to show that if $(x^+,x^-_{\widehat{I}})\in R$ then $(x^+,x^-_{\widehat{I\cup \{j\}}})\in R$ for any $j\notin I$. But this follows since $(x^-_je_j,{\mathbb {0}})\in R$ and since

$$\begin{aligned} (x^+,x^-_{\widehat{I\cup \{j\}}})=(x^+,x^-_{\widehat{I}})\oplus _j (x^-_je_j,{\mathbb {0}}) \in R. \end{aligned}$$

In this way, by induction (successively increasing *I* to $[n]\backslash \{i\}$), we conclude that that $(x^+,x^-_ie_i)\in R$ for all $i\in \operatorname {supp}(x^-)$.

We also observe that $(x^+,y^-_i e_i)\in R$, as for the case when $y^-_i\ne -\inf$ we have that $x_i^-\geqslant y_i^-$ and $(x^+,y^-_i e_i)=(x^-_i)^{-1} y^-_i (x^+,x^-_ie_i)\oplus (x^+,{\mathbb {0}})$.

It follows that $(x^+,y^-_ie_i)\in R$ for all $i\in \operatorname {supp}(y^-)$, and then $(x^+,y^-)\in R$ as $(x^+,y^-)=\bigoplus _{i=1}^n (x^+,y^-_ie_i)$.

Finally, $$(y^+,y^-)=(x^+,y^-) \oplus (y^+,{\mathbb {0}})\in R$$. $$\square $$

#### Lemma 3.3

Let *R* be a signed elimination cone. Then, $x,y\in R$ implies $(x\,{\widehat{\oplus }}\,y)^{\vee }\in R$.

#### Proof

We introduce vectors *u*, *v*, *z* such that $u\oplus z=x^-$, $v\oplus z = y^+$ and supports of *v* and *z*, as well as the supports of *u* and *z*, are pairwise disjoint. In this new notation $(x\,{\widehat{\oplus }}\,y)^{\vee }=(x^+\oplus v, u\oplus y^-)^{\vee }$.

We will prove by induction that $(x^+\oplus v\oplus z_{\widehat{I}},u\oplus y^-)^{\vee }\in R$ for all $I\subseteq [n]$. The basis of induction is $(x^+\oplus v\oplus z,u\oplus y^-)^{\vee }\in R$, which is equal to $((x^+,u)\oplus (v\oplus z,y^-))^{\vee }$. Here $(x^+,u)\in R$ by Lemma 3.2.

We need to show that if $(x^+\oplus v\oplus z_{\widehat{I}},u\oplus y^-)^{\vee }\in R$ then $(x^+\oplus v\oplus z_{\widehat{I\cup \{j\}}},u\oplus y^-)^{\vee }\in R$ for any $j\notin I$. For this, first note that $(x^+,u\oplus z_{\widehat{I}})\in R$ by Lemma 3.2, and that all components of $z_{\widehat{I}}$ are present in $(x^+\oplus v\oplus z_{\widehat{I}},u\oplus y^-)^{\vee }$ since the supports of *z* and *u* are disjoint by the definition of these vectors and the supports of *z* and $y^-$ are disjoint since $(v\oplus z,y^-)\in R$. Then we observe that

$$\begin{aligned} \begin{aligned}&\left( (x^+,u\oplus z_{\widehat{I}})\oplus _j (x^+\oplus v\oplus z_{\widehat{I}},u\oplus y^-)^{\vee }\right) ^{\vee }\\&\quad = (x^+\oplus v\oplus z_{\widehat{I\cup \{j\}}},u\oplus y^-\oplus z_{\widehat{I\cup \{j\}}})^{\vee }=\\&\quad = (x^+\oplus v\oplus z_{\widehat{I\cup \{j\}}},u\oplus y^-)^{\vee }. \end{aligned} \end{aligned}$$

Therefore $(x^+\oplus v\oplus z_{\widehat{I\cup \{j\}}},u\oplus y^-)^{\vee }\in R$, as claimed.

Setting $I=[n]$ we obtain $(x^+\oplus v, u\oplus y^-)^{\vee }\in R$. As $(x^+\oplus v, u\oplus y^-)^{\vee }=(x\,{\widehat{\oplus }}\,y)^{\vee }$, the proof is complete. $\square$

#### Proposition 3.3

Suppose that $R\subset ({\mathbb {T}}^n)^2$ consists of signed vectors and satisfies (i) and (ii) of Definition 3.10. Then the conditions (iii) and (iv) of this definition hold if and only if *R* is stable under the $\,{\widehat{\oplus }}^\vee \,$ operation.

#### Proof

The “only if” part follows from Lemma 3.3.

Conversely, using Lemma 3.2, it suffices to note that $(x\,{\widehat{\oplus }}\,y)^\vee \preccurlyeq (x \oplus _i y)^\vee \preccurlyeq (x \oplus y)^\vee$, which follows using (7) by comparing the positive parts (the negative parts remain the same). $\square$

#### Remark 3.4

The above result means that $R\subset ({\mathbb {T}}^n)^2$ is a signed elimination cone if and only if it contains ${\mathbb {T}}^n$ and is a convex cone with respect to the $\odot$ scalar multiplication and $\,{\widehat{\oplus }}^\vee \,$ addition.

#### Proposition 3.4

Let $R\in ({\mathbb {T}}^n)^2$ be a signed elimination cone and define $C=R\oplus \varDelta ^n$. Then *C* is a monotone pre-congruence.

#### Proof

We have to prove that *C* satisfies all the properties listed in Definition 3.3.

Definition 3.3, (iv): For $(f^+,f^-)$ such that $f^+\geqslant f^-$ we can write $(f^+,f^-)=(f^+,{\mathbb {0}})\oplus (f^-,f^-)$. By Definition 3.10 (i) we have $(f^-,{\mathbb {0}})\in R$, hence $(f^+,f^-)\in C$.

Definition 3.3, (i): Follows from Definition 3.10 (ii).

Definition 3.3 (ii): We have

$$\begin{aligned} (f^+,f^-)\oplus (g^+,g^-)=((f^+,f^-)^{\vee }\oplus (g^+,g^-)^{\vee })^{\vee }\oplus (d,d). \end{aligned}$$

where $d_i=\min (f^+_i\oplus g^+_i,\; f^-_i\oplus g^-_i)$ for all *i*. It suffices to check this property for the case when $f^+,f^-,g^+$ and $g^-$ are scalars, which is elementary. It follows then that $(f^+,f^-)\oplus (g^+,g^-)\in C$ since $(f^+,f^-)^{\vee }\in R$ and $(g^+,g^-)^{\vee }\in R$ (note that if we represent $(f^+,f^-)=(x^+,x^-)\oplus (c,c)$ with $(x^+,x^-)\in R$ then $(f^+,f^-)^{\vee }\succcurlyeq (x^+,x^-)$).

Definition 3.3, (iii): Let us represent

$$\begin{aligned} (f^+,f^-)=(x^+,x^-)\oplus (c,c),\quad (g^+,g^-)=(y^+,y^-)\oplus (d,d), \end{aligned}$$

where $$(x^+,x^-)\in R$$, $$(y^+,y^-)\in R$$ and $$f^-=g^+$$.

Denote by *I* the set of indices for which $x^-_i=f^-_i$. Then we can write

$$\begin{aligned} (f^+,f^-)=(x^+,f^-_I)\oplus (c,c), \end{aligned}$$

where $(x^+,f^-_I)\in R$ by Lemma 3.2 (since $(x^+,x^-)\in R$ and $x^-\geqslant f^-_I$). We can also write

$$\begin{aligned} (g^+,g^-)=(y^+\oplus d,y^-)^{\vee }\oplus (d,d)=(g^+_J,z)\oplus (d,d) \end{aligned}$$

for some index set $J\subseteq [n]$ and some *z* such that $(y^+\oplus d,y^-)^{\vee }=(g^+_J,z)$ (it is easy to check that the nonzero components of the positive part of $(y^+\oplus d,y^-)^{\vee }$ are components of $g^+$). Here we also have that $(g^+_J,z)=(y^+\oplus d,y^-)^{\vee }=((y^+,y^-)\oplus (d,{\mathbb {0}}))^{\vee }$ is in *R* by Definition 3.10 (iii). We then can write

8 $$\begin{aligned} (f^+,f^-)=(x^+,f^-_I)\oplus (c,c),\quad (g^+,g^-)=(g^+_J,z)\oplus (d,d) \end{aligned}$$

and denote $K=I\cap J$ and $L=\widehat{I\cup J}$ (the complement of $I\cup J$).

By Lemma 3.3 we obtain $((x^+,f^-_I)\,{\widehat{\oplus }}^\vee \,(g^+_J,z))\in R$, hence

$((x^+,f^-_I)\,{\widehat{\oplus }}\,(g^+_J,z)) = (x^+\oplus g^+_{J\backslash I}, f^-_{I\backslash J}\oplus z)\in C$. From (8) and $f^-=g^+$, we have $f^-_I\oplus c=g^+_J\oplus d$, and therefore

9 $$\begin{aligned} c_{I\backslash J}\oplus f^-_{I\backslash J}=d_{I\backslash J},\quad c_{J\backslash I}=d_{J\backslash I}\oplus g^+_{J\backslash I}, \end{aligned}$$

and

10 $$\begin{aligned} c_K\oplus f^-_K=d_K\oplus g^+_K,\quad c_L=d_L. \end{aligned}$$

Then Equation (9) together with $C\oplus \varDelta ^n\subset C$ implies that

$$\begin{aligned} \begin{aligned}&(x^+\oplus g^+_{J\backslash I}, f^-_{I\backslash J}\oplus z)\oplus (c_{I\backslash J}\oplus d_{J\backslash I} ,c_{I\backslash J} \oplus d_{J\backslash I})\\&\quad =(x^+\oplus c_{I\backslash J}\oplus c_{J\backslash I}, z\oplus d_{I\backslash J}\oplus d_{J\backslash I})\in C. \end{aligned} \end{aligned}$$

Equation (10) implies that for any $i\in K$ we have either $c_i=d_i>f^-_i(=g^+_i)$ or $c_i\leqslant f^-_i(=g^+_i)$ and $d_i\leqslant f^-_i(=g^+_i)$, and that we have $c_i=d_i$ for any $i\in L$. Define the sets

$$\begin{aligned} K_1= \{i\in K :c_i=d_i>f^-_i\}\cup L,\quad K_2=\{i\in K:c_i\leqslant f^-_i\; \text { and }\; d_i\leqslant f^-_i\}. \end{aligned}$$

Note that $I\backslash J\cup J\backslash I\cup K_1\cup K_2=[n]$, and that we have $c_{K_1}=d_{K_1}$. Also as $(x^+, f^-_I)\in R$ and $f^-_I\geqslant f^-_{K_2}\geqslant d_{K_2}$, we obtain that $(x^+,d_{K_2})\in R$ by Lemma 3.2.

We then show, using Equation (8) and the facts established above, that $(f^+,g^-)\in C$ since

$$\begin{aligned} \begin{aligned} (f^+, g^-)&= (x^+\oplus c, z\oplus d)\\&= (x^+\oplus c_{I\backslash J}\oplus c_{J\backslash I}\oplus c_{K_1}\oplus c_{K_2}, z\oplus d_{I\backslash J}\oplus d_{J\backslash I}\oplus d_{K_1}\oplus d_{K_2})\\&=(x\oplus c_{I\backslash J}\oplus c_{J\backslash I}, z\oplus d_{I\backslash J}\oplus d_{J\backslash I})\oplus (c_{K_1},c_{K_1})\\&\qquad \oplus (c_{K_2},0)\oplus (x^+,d_{K_2}). \end{aligned} \end{aligned}$$

This completes the proof. $\square$

#### Lemma 3.4

Let $U \subset ({\mathbb {S}}^{\vee })^n$ and $R:=\imath (U)\subset ({\mathbb {T}}^n)^2$. Then, *U* is closed in the product of the order topology on ${\mathbb {S}}^{\vee }$ if and only if *R* is closed in the topology induced by the Euclidean topology on $({\mathbb {T}}^n)^2$.

#### Proof

The Euclidean topology on $({\mathbb {T}}^n)^2$ is defined as a product topology, and $({\mathbb {S}}^{\vee })^n$ is also equipped with a product topology, so it suffices to show the result when $n=1$. Then it is elementary to verify that the order topology on ${\mathbb {S}}^{\vee }$ coincides with the topology induced by the Euclidean topology on the set of signed pairs of ${\mathbb {T}}^2$ – indeed, these are two realizations of the same topological space, obtained by gluing two copies of the half-line $[-\infty ,\infty )$ (equipped with the order topology) at point $-\infty$. $\square$

#### Proof of Theorem 3.2

(2)$\Rightarrow$(1). If *C* is a closed monotone pre-congruence, then the signed part of *C*, $R=C^{\vee }$, is a signed elimination cone, by Proposition 3.2. Moreover, observe that the subset of signed pairs of $({\mathbb {T}}^n)^2$ is closed. Hence, $C^{\vee }$, which is the intersection of the closed set *C* with this subset, is also closed.

(1)$\Rightarrow$(2). If *R* is a signed elimination cone, then, by Proposition 3.4, $C= R\oplus \varDelta ^n$ is a monotone pre-congruence. We have also that *R* is the signed part of *C*. Moreover, suppose that *R* is closed. Let $c_k = r_k \oplus \delta _k$ with $r_k\in R$ and $\delta _k\in \varDelta ^n$ be a sequence of elements of *C* converging to $c\in ({\mathbb {T}}^n)^2$. Then the sequences $r_k$ and $\delta _k$ are bounded from above. By taking subsequences, we may assume that $r_k$ converges to some *r* and that $\delta _k$ converges to some $\delta$. Since $\varDelta ^n$ and *R* are closed, we deduce that $c=r \oplus \delta \in C$. So, *C* is closed. $\square$

#### Proof of Theorem 3.3

(2)$\Rightarrow$(1). If $U=A^\circ$ for some subset $A\subset {\mathbb {T}}^n$, then, by (6), $R:=\imath (U)$ is the signed part of the polar $A^\triangleright$. By Theorem 3.1, $A^\triangleright$ is a closed monotone pre-congruence. Hence, by Theorem 3.2, (2) $\Rightarrow$ (1), *R* is a signed elimination cone that is closed in the topology of $({\mathbb {T}}^n)^2$, i.e., the Euclidean topology. Then, by Lemma 3.4, $\imath (R)\subset ({\mathbb {S}}^{\vee })^n$ is closed under the product order topology on $({\mathbb {S}}^{\vee })^n$.

(1)$\Rightarrow$(2). Suppose now that $\imath (U)$ is a signed elimination cone, and that *U* is closed in $({\mathbb {S}}^{\vee })^n$. Then, using again Lemma 3.4, we get that $\imath (U)$ is closed in the Euclidean topology of $({\mathbb {T}}^n)^2$. Then, by Theorem 3.2, (1) $\Rightarrow$ (2), $\imath (U)$ is the signed part of a closed monotone pre-congruence *C* of ${\mathbb {T}}^n$. Then, by Theorem 3.1, we have $C=A^\triangleright$ where $A=C^\triangleleft$.

Hence, $\imath (U)$ is the set of signed couples $(x^+,x^-) \in ({\mathbb {T}}^n)^2$ such that $\langle x^+, a\rangle \geqslant \langle x^-,a\rangle$ holds for all $a\in A$. By (6), this means that *U* is the set of vectors $x\in ({\mathbb {S}}^{\vee })^n$ such that $\langle x,a\rangle \succcurlyeq {\mathbb {0}}$ for all $a\in A$, i.e., $U=A^\circ$. $\square$

### Valuation of Polars

In this subsection we relate signed polars to images by valuation of classical polars. Let us denote by $\operatorname {cl}$ the closure of a set and by $\operatorname {int}$ its interior. We use the product of order topologies, both on $({\mathbb {S}}^{\vee })^n$ and on ${\mathbb {K}}^n$. Let us first observe the following useful property of tropical polars.

#### Lemma 3.5

Let $$A\subset {\mathbb {T}}^n$$. Then $$\operatorname {cl}\operatorname {int}(A^{\circ })=A^{\circ }$$.

#### Proof

Let $x\in A^{\circ }$. Denote $N_+=\{i:x_i\succ {\mathbb {0}}\}$, $N_-=\{i:x_i\prec {\mathbb {0}}\}$ and $N_0=\{i:x_i={\mathbb {0}}\}$. Using the canonical representation $x=x^+\ominus x^-$ where both $x^+$ and $x^-$ are positive (in the sense of symmetrization) vectors with disjoint support, we define vectors $z^k\in ({\mathbb {S}}^{\vee })^n$ for $k\geqslant 1$ as follows:

$$\begin{aligned} z^k_i= {\left\{ \begin{array}{ll} t_k+ x_i^+, & \text { if } i\in N_+,\\ \ominus x_i^-, & \text { if } i\in N_-,\\ M_k, & \text { if } i\in N_0, \end{array}\right. } \end{aligned}$$

where $M_k\succ {\mathbb {0}}$ (positive in the sense of symmetrization) and $t_k>0$ (a real number, positive in the usual sense). Vectors $z^k$ can be chosen in such a way that $\lim _{k\rightarrow \infty } z^k=x$ in the product order topology. For example, we can choose $M_k=\log \epsilon _k$ where $\epsilon _k>0$ and $t_k$ satisfying $\epsilon _k=(e^{t_k}-1)\cdot \max _{i\in N_+} e^{x_i^+}$, with $\lim _{k\rightarrow \infty } \epsilon _k=0$. We then see that $e^{M_k}=\epsilon _k$ and $|e^{z^{k+}_i}-e^{x_i^+}|\leqslant \epsilon _k$, ensuring that $\lim _{k\rightarrow \infty } z^k=x$.

Now fix $t'_k>0$ such that $t'_k<t_k$, and consider $y\in ({\mathbb {S}}^{\vee })^{n}$ with canonical representation $y^+\ominus y^-$ that belongs to the open neighborhood of $z^k$ consisting of vectors *y* such that

11 $$\begin{aligned} \begin{aligned}&\text { for } i\in N_-:\quad {\mathbb {0}}\succ y_i \succ \ominus (x_i^-+t'_k),\\&\quad \text { for } i\in N_+:\quad y_i\succ x_i^++t'_k,\\&\quad \text { for } i\in N_0:\quad y_i\succ {\mathbb {0}}. \end{aligned} \end{aligned}$$

Let $a\in A$, then, using properties (11), we obtain

$$\begin{aligned} \langle y^+,a\rangle \geqslant \bigoplus _{i\in N_0} y_i^+\odot a_i\oplus \bigoplus _{i\in N_+} y_i^+\odot a_i\geqslant \bigoplus _{i\in N_-} y_i^-\odot a_i=\langle y^-,a\rangle , \end{aligned}$$

as it can be argued that

$$\begin{aligned} \bigoplus _{i\in N_+} y_i^+\odot a_i\geqslant t'_k+ \bigoplus _{i\in N_+} x_i^+\odot a_i\geqslant t'_k +\bigoplus _{i\in N_-} x_i^-\odot a_i\geqslant \bigoplus _{i\in N_-} y_i^-\odot a_i. \end{aligned}$$

We thus obtain $y\in A^{\circ }$ for any such *y*, implying that $z^k\in \operatorname {int}(A^{\circ })$. As $\lim _{k\rightarrow \infty } z^k=x$, the claim follows. $\square$

The following theorem is the main result of this subsection.

#### Theorem 3.4

Let $\varvec{A}$ be a closed semialgebraic subset of ${\mathbb {K}}_{\geqslant 0}^n$. Then,

12 $$\begin{aligned} \operatorname {sval}(\varvec{A}^{\circ })= (\operatorname {val}\varvec{A})^{\circ }. \end{aligned}$$

The proof of Theorem 3.4 relies on auxiliary results.

#### Lemma 3.6

Let *A* be a subset of ${\mathbb {T}}^n$ and let $x=x^+\ominus x^-$ belong to $\operatorname {int}A^{\circ }$. Then

$$\begin{aligned} \langle x^+,a\rangle >\langle x^-,a\rangle ,\quad \forall a\in A\backslash \{{\mathbb {0}}\}. \end{aligned}$$

#### Proof

By contradiction, suppose that there exists $a\in A\backslash \{{\mathbb {0}}\}$ such that $\langle x^+,a\rangle =\langle x^-,a\rangle$.

Suppose first that the latter value is equal to ${\mathbb {0}}$, and let $i\in [n]$ be such that $a_i \ne {\mathbb {0}}$. Let $e_i$ denote the *i*th unit vector of ${\mathbb {S}}^n$, consider $y= x\ominus t e_i$, with *t* positive. Then, $y_j = x_j$ holds for all $j\ne i$ and $y_i = \ominus t$. It follows that $\langle y^+,a\rangle ={\mathbb {0}}< \langle y^-,a\rangle = t a_i$. This contradicts that *x* belongs to $\operatorname {int}A^{\circ }$.

Suppose now that $\langle x^+,a\rangle =\langle x^-,a\rangle \ne {\mathbb {0}}$. Then there exists $t\in {\mathbb {T}}$ such that $t>\mathbbm {1}$ and $y=x^+\ominus t x^-$ still belongs to $A^{\circ }$. However, we have $\langle y^+,a\rangle <\langle y^-,a\rangle$ in contradiction with $y\in A^{\circ }$, and this proves the claim. $\square$

We deduce the following.

#### Lemma 3.7

$$\operatorname {int}((\operatorname {val}\varvec{A})^{\circ })\subset \operatorname {sval}(\varvec{A}^{\circ })$$.

#### Proof

Take $x\in \operatorname {int}((\operatorname {val}\varvec{A}))^{\circ })$ and consider $\varvec{x}$ with coordinates defined by $\varvec{x}_i=\operatorname {sgn}(x_i)t^{|x_i|}$. Then for each $\varvec{a}\in \varvec{A}$ we have $\langle x^+,\operatorname {val}(\varvec{a})\rangle >\langle x^-,\operatorname {val}(\varvec{a})\rangle$ by Lemma 3.6 and therefore also $\langle \varvec{x},\varvec{a}\rangle \geqslant 0$ for the lift. This shows $x\in \operatorname {sval}(\varvec{A}^{\circ })$. $\square$

We are now ready to complete the proof of the main statement.

#### Proof of Theorem 3.4

We first prove the inclusion $\operatorname {sval}(\varvec{A}^{\circ })\subset (\operatorname {val}\varvec{A})^{\circ }.$ For each $x\in \operatorname {sval}(\varvec{A}^{\circ })$ and $\varvec{x}$ such that $x=\operatorname {sval}(\varvec{x})$ we have that $\langle \varvec{x},\varvec{a}\rangle \geqslant \varvec{0}$ for all $\varvec{a}\in \varvec{A}$, which we can write as $\langle \varvec{x^+},\varvec{a}\rangle \geqslant \langle \varvec{x^-},\varvec{a}\rangle$. Taking valuation of this we obtain $\langle x^+,\operatorname {val}(\varvec{a})\rangle \geqslant \langle x^-,\operatorname {val}(\varvec{a})\rangle$ for each $\varvec{a}\in \varvec{A}$, which shows the inclusion.

Now, combining (12) and Lemma 3.7 we obtain

13 $$\begin{aligned} \operatorname {int}((\operatorname {val}\varvec{A})^{\circ })\subset \operatorname {sval}(\varvec{A}^{\circ })\subset (\operatorname {val}\varvec{A})^{\circ }. \end{aligned}$$

We recall that if ${\textbf{B}}$ is a closed semi-algebraic subset of ${\mathbb {K}}^n$, then, $\operatorname {sval}({\textbf{B}})$ is a closed subset of $({\mathbb {S}}^{\vee })^n$, this follows from Theorem 6.9 of [40] or alternatively from of Corollary 4.11 of [12]. Hence, applying the topological closure to the chain of inclusions (13), and using that: 1) $\operatorname {cl}\operatorname {int}((\operatorname {val}\varvec{A})^{\circ })= (\operatorname {val}\varvec{A})^{\circ }$ by Lemma 3.5, 2) $\operatorname {sval}(\varvec{A}^{\circ })$ is closed by the above property; 3) $(\operatorname {val}\varvec{A})^{\circ }$ is trivially closed (being a tropical polar), we obtain the “sandwich”

$$\begin{aligned} (\operatorname {val}\varvec{A})^{\circ }\subset \operatorname {sval}(\varvec{A}^{\circ })\subset (\operatorname {val}\varvec{A})^{\circ }, \end{aligned}$$

from which the claim follows. $\square$

#### Remark 3.5

We know from [12] that if ${\textbf{A}}$ is a closed semi-algebraic subset of ${\mathbb {K}}_{\geqslant 0}^n$, then, the subset of $\operatorname {val}({\textbf{A}})$ with a given support is a closed semilinear set. Moreover, this subset can be computed from a definition of ${\textbf{A}}$ by polynomial inequalities, under a genericity condition, see [12, Coro 4.8].

## Tropicalization of Matrix Cones and their Polars

### Tropical Positive Definite Matrices and Completely Positive Matrices

We say that a symmetric matrix $A\in ({\mathbb {S}}^\vee )^{n\times n}$ is a *signed tropical positive semidefinite matrix* if $x^T Ax \succcurlyeq 0$ for all $x\in ({\mathbb {S}}^\vee )^n$.

We denote by $\operatorname {PSD}_n({\mathbb {S}})$ the set of signed tropical positive semidefinite matrices and denote by $\operatorname {PSD}_n({\mathbb {T}}) = \operatorname {PSD}_n({\mathbb {S}})\cap {\mathbb {T}}^{n\times n}$ the set of *tropical positive semidefinite matrices*. We first examine the special case of $2\times 2$ matrices.

#### Lemma 4.1

Let $$a,b,c\in {\mathbb {S}}^\vee $$. Then,

$$\begin{aligned} a x_1^2 \oplus bx_1x_2 \oplus cx_2^2 \succcurlyeq {\mathbb {0}},\; \forall x_1,x_2\in {\mathbb {S}}^\vee \end{aligned}$$

holds if and only if $a\succcurlyeq 0,\; c\succcurlyeq {\mathbb {0}}$ and $b^2 \preccurlyeq ac.$

#### Proof

“Only if”. By taking $x_1 =\mathbbm {1}$ and $x_2={\mathbb {0}}$, we see that the condition $a\succcurlyeq {\mathbb {0}}$ is necessary. Similarly, $c\succcurlyeq {\mathbb {0}}$ is necessary. If $a={\mathbb {0}}$, then, by taking $x_2=\mathbbm {1}$ and $x_1 = \ominus ub$ with $u\in {\mathbb {T}}$ large enough, we get $a x_1^2 \oplus bx_1x_2 \oplus cx_2^2 = \ominus ub^2 \oplus c\succcurlyeq {\mathbb {0}}$ which holds for all such *u* only if $b={\mathbb {0}}$. Then the condition $b^2\preccurlyeq ac$ trivially holds. By symmetry, the same is true if $c={\mathbb {0}}$. We may now assume that *a*, *c* are positive elements of ${\mathbb {S}}^\vee$. Then, taking $x_1 =\mathbbm {1}/\sqrt{a}$ and $x_2 = \epsilon \mathbbm {1}/\sqrt{c}$ with $\epsilon \in \{\mathbbm {1},\ominus \mathbbm {1}\}$, we get $\mathbbm {1}\oplus \epsilon b/(\sqrt{ac})\succcurlyeq {\mathbb {0}}$, which is possible only if $|b|/\sqrt{ac}\preccurlyeq \mathbbm {1}$, or equivalently $b^2\preccurlyeq ac$.

“If”. The case in which $a={\mathbb {0}}$ or $c={\mathbb {0}}$ is trivial, so, we suppose that *a* and *c* are both positive, and make the change of variable $x_1=y_1/\sqrt{a}$ and $x_2=y_2/\sqrt{c}$. Then, $a x_1^2 \oplus bx_1x_2 \oplus cx_2^2\succcurlyeq {\mathbb {0}}$ holds if and only if

14 $$\begin{aligned} y_1^2 \oplus u y_1y_2 \oplus y_2^2 \succcurlyeq {\mathbb {0}}, \end{aligned}$$

where $u:= b/(\sqrt{a}\sqrt{c})$ is such that $|u|\preccurlyeq \mathbbm {1}$. Without loss of generality, we may assume that $y_1^2\succcurlyeq y_2^2$. Then (14) can be rewritten as $y_1^2 \oplus uy_1y_2\succcurlyeq {\mathbb {0}}$, which holds since $|uy_1y_2|= |u|\sqrt{|y_1|^2 |y_2|^2} \preccurlyeq |u||y_1|^2 \preccurlyeq y_1^2$. $\square$

The condition $b^2\preccurlyeq ac$ in Lemma 4.1 is the tropical analogue of the nonpositivity of the discriminant.

#### Theorem 4.1

$$\begin{aligned} \begin{aligned} \operatorname {PSD}_n({\mathbb {S}})&=\{ A \in ({\mathbb {S}}^\vee )^{n\times n} \mid A_{ii}\succcurlyeq {\mathbb {0}}, \forall i\in [n],\\ A_{ij}&=A_{ji},\; A_{ij}^2 \preccurlyeq A_{ii}A_{jj}, \forall i,j\in [n], i\ne j\}. \end{aligned} \end{aligned}$$

In other words, $\operatorname {PSD}_n({\mathbb {S}})$ consists of those matrices $A\in ({\mathbb {S}}^\vee )^{n\times n}$ such that $A^-_{ii}={\mathbb {0}}$ for all $i\in [n]$ and $(A_{ij}^\pm )^2 \preccurlyeq A_{ii}^+ A_{jj}^+$ for $i,j\in [n]$ with $i\ne j$.

#### Proof

“Only if”. This is obtained from Lemma 4.1, by noting that if $A\in ({\mathbb {S}}^\vee )^{n\times n}$, then any $2\times 2$ principal submatrix of *A* must be in $\operatorname {PSD}_2({\mathbb {S}})$.

“If”. If *A* satisfies the condition of the theorem, by Lemma 4.1, we have $x_{i}A_{ii}x_i \oplus x_j A_{jj}x_j \oplus x_i A_{ij}x_j\succcurlyeq {\mathbb {0}}$ for all $i\ne j$. By summing these inequalities over all $i\ne j$, we arrive at $x^{T}Ax\succcurlyeq {\mathbb {0}}$. $\square$

We have the following immediate corollary.

#### Corollary 4.1

$$\begin{aligned} \operatorname {PSD}_n({\mathbb {T}}) =\{ A \in {\mathbb {T}}^{n\times n} \mid \;A_{ij}=A_{ji},\; A_{ij}^2 \preccurlyeq A_{ii}A_{jj}, \forall i,j\in [n], i\ne j\}. \end{aligned}$$

A matrix $X\in {\mathbb {T}}^{n\times n}$ is called *tropical completely positive of order k* (where $k\geqslant 1$) if it has entries

$$\begin{aligned} X_{ij}= \langle y^i,y^j\rangle \hspace{5.0pt},\quad i,j\in [n], \end{aligned}$$

for some vectors $y^i\in {\mathbb {T}}^k$ (for $i\in [n]$), where $\langle x,y\rangle :=\bigoplus _i x_i \odot y_i$ denotes the canonical scalar product on ${\mathbb {T}}^k$. The set of all such matrices *X* for a fixed *k* is denoted by $\operatorname {CP}_{n,k}({\mathbb {T}})$.

A matrix $X\in {\mathbb {T}}^{n\times n}$ is called *tropical completely positive semidefinite of order k* (where $k\geqslant 1$) if it has entries

$$\begin{aligned} X_{ij}= \langle Y^i,Y^j\rangle \hspace{5.0pt},\quad i,j\in [n], \end{aligned}$$

for some $Y^i\in \operatorname {PSD}_k({\mathbb {T}})$ (for $i\in [n]$), where the Frobenius scalar product $\langle \cdot , \cdot \rangle$ is understood in the tropical sense, i.e., $\langle X, Y\rangle :=\bigoplus _{ij}X_{ij}\odot Y_{ij}$. The set of all such matrices *X* for a fixed *k* is denoted by $\operatorname {CPSD}_{n,k}({\mathbb {T}})$.

The sets of tropical completely positive matrices and, respectively, tropical completely positive definite matrices are $\operatorname {CP}_n({\mathbb {T}})=\cup _{k\geqslant 1} \operatorname {CP}_{n,k}({\mathbb {T}})$ and, respectively, $\operatorname {CPSD}_n({\mathbb {T}})=\cup _{k\geqslant 1}\operatorname {CPSD}_{n,k}({\mathbb {T}})$. These sets are also referred to as *tropical completely positive cone* and *tropical completely positive semidefinite cone.* In what follows for a square matrix *X* we denote by $\operatorname {diag}(X)$ the vector of diagonal entries of *X*.

#### Lemma 4.2

For all $$Y,Z\in \operatorname {PSD}_n({\mathbb {T}})$$,

$$\begin{aligned} \langle Y, Z \rangle = \langle \operatorname {diag}(Y),\operatorname {diag}(Z)\rangle . \end{aligned}$$

#### Proof

We have $\langle Y, Z \rangle =\bigoplus _{ij}Y_{ij}Z_{ij} \preccurlyeq \bigoplus _{ij}\sqrt{Y_{ii}Y_{jj}}\sqrt{Z_{ii}Z_{jj}}$. Observe that

$$\begin{aligned} \sqrt{Y_{ii}Y_{jj}}\sqrt{Z_{ii}Z_{jj}}= \sqrt{Y_{ii}Z_{ii}} \sqrt{Y_{jj}Z_{jj}} \preccurlyeq Y_{ii}Z_{ii} \oplus Y_{jj}Z_{jj} \preccurlyeq \langle \operatorname {diag}(Y),\operatorname {diag}(Z)\rangle , \end{aligned}$$

showing that $\langle Y,Z\rangle \preccurlyeq \langle \operatorname {diag}(Y),\operatorname {diag}(Z)\rangle$. The other inequality is trivial. $\square$

#### Remark 4.1

If $Y,Z\in \operatorname {PSD}_n({\mathbb {S}})$, then (in general) we only get

$$\langle Y,Z \rangle \nabla \langle \operatorname {diag}(Y),\operatorname {diag}(Z)\rangle $$ and $$|\langle Y,Z \rangle | $$ $$=| \langle \operatorname {diag}(Y),\operatorname {diag}(Z)\rangle |$$.

We note that the two classes of matrices $\operatorname {CPSD}_n$ and $\operatorname {CP}_n$ coincide in the tropical setting.

#### Proposition 4.1

$$\operatorname {CPSD}_{n,k}({\mathbb {T}})=\operatorname {CP}_{n,k}({\mathbb {T}})$$.

#### Proof

If $X\in \operatorname {CP}_{n,k}({\mathbb {T}})$, then $X_{ij}=(y^i)^Ty^j$ for some vectors $y^i\in {\mathbb {T}}^k$, and so $X_{ij} =\langle \operatorname {diag}(y^i),\operatorname {diag}(y^j)\rangle \in \operatorname {CPSD}_{n,k}({\mathbb {T}})$. Conversely, if $X\in \operatorname {CPSD}_{n,k}({\mathbb {T}})$, we have $X_{ij}=\langle Y^i,Y^j \rangle$ for some $Y^i\in \operatorname {PSD}_k({\mathbb {T}})$, and so, by Lemma 4.2, $X_{ij}=(y^i)^Ty^j$ where $y^i=\operatorname {diag}(Y^i)$, which implies that $X\in \operatorname {CP}_{n,k}({\mathbb {T}})$. $\square$

#### Remark 4.2

Recall that the cp-rank of a $n\times n$ matrix $\varvec{A}$ is the smallest integer *k* such that $\varvec{A}\in \operatorname {CP}_{n,k}({\mathbb {K}})$. The csdp-rank of $\varvec{A}$ is the smallest integer *k* such that $\varvec{A}\in \operatorname {CPSD}_{n,k}({\mathbb {K}})$. Proposition 4.1 entails that the tropical analogues of these two notions of rank coincide.

#### Theorem 4.2

$$\begin{aligned} \begin{aligned}&\operatorname {sval}(\operatorname {PSD}_n({\mathbb {K}})) = \operatorname {PSD}_n({\mathbb {S}}),\\&\quad \operatorname {val}(\operatorname {PSD}_n({\mathbb {K}})\cap {\mathbb {K}}_{\geqslant 0}^{n\times n}) = \operatorname {val}(\operatorname {PSD}_n({\mathbb {K}})) = \operatorname {PSD}_n({\mathbb {T}}). \end{aligned} \end{aligned}$$

#### Proof

We prove the first of these equalities. The inclusion $\operatorname {sval}(\operatorname {PSD}_n({\mathbb {K}}))\subseteq \operatorname {PSD}_n({\mathbb {S}})$ is obvious, because for each $\varvec{A}\in \operatorname {PSD}_n({\mathbb {K}})$ we have $\varvec{A}_{ii},\varvec{A}_{jj}\geqslant 0$ and $\varvec{A}_{ij}^2\leqslant \varvec{A}_{ij}\varvec{A}_{ji}$.

Let us prove the other inclusion. Take $A\in \operatorname {PSD}_n({\mathbb {S}})$ and also define matrix $\varvec{B}$ with entries

$$\begin{aligned} \varvec{b}_{ij}= {\left\{ \begin{array}{ll} (n-1), & \text { if } i=j,\\ 1, & \text { if } i\ne j. \end{array}\right. } \end{aligned}$$

Now let $\varvec{C}$ be the lift of *A*, with entries $\varvec{c}_{ij}=\epsilon _{ij} \varvec{b}_{ij} t^{|A_{ij}|}$ and $\epsilon _{ij}=\operatorname {sgn}(A_{ij})$. Then $\varvec{C}\in \operatorname {PSD}_n({\mathbb {K}})$ by [12], Lemma 5.4.

The proof of the other equalities is similar and uses $\varvec{c}_{ij}= \varvec{b}_{ij} t^{|A_{ij}|}$ $\square$

#### Corollary 4.2

([58]) The set $\operatorname {val}(\operatorname {PSD}_n({\mathbb {K}})\cap ({\mathbb {K}}^*)^{n\times n})$ coincides with the set of matrices $A=(A_{ij})\in {\mathbb {R}}^{n\times n}$ such that $2 A_{ij}\leqslant A_{ii}+A_{jj}$.

Let us recall the following result of Cartwright and Chan.

#### Theorem 4.3

([20, Proposition 2 and Theorem 4]) We have, for all $k\geqslant \max (n,\lfloor n^2/4\rfloor )$,

$$\begin{aligned} \operatorname {CP}_n({\mathbb {T}})=\operatorname {CP}_{n,k}({\mathbb {T}}) = \{M\in {\mathbb {T}}^{n\times n}\mid M_{ij}=M_{ji},\ M_{ij}^{2}\leqslant M_{ii} M_{jj}\ \forall i,j\}. \end{aligned}$$

Together with Corollary 4.1 and Proposition 4.1, this implies the following result:

#### Corollary 4.3

$$\operatorname {CP}_n({\mathbb {T}}) = \operatorname {PSD}_n({\mathbb {T}}) = \operatorname {CPSD}_n({\mathbb {T}}).$$

We also make the following observation:

#### Proposition 4.2

$$\operatorname {val}(\operatorname {CP}_n({\mathbb {K}}))=\operatorname {CP}_n({\mathbb {T}}).$$

#### Proof

Indeed, consider a tropical matrix of the form $X=Y Y^T$. Then we can take the monomial lift $\varvec{Y}_{ij}=t^{Y_{ij}}$, and then $\operatorname {val}(\varvec{Y}\varvec{Y}^T)=Y Y^T=X$ by the properties of valuation over ${\mathbb {K}}_{\geqslant 0}$. This shows $\operatorname {CP}_n({\mathbb {T}})\subseteq \operatorname {val}(\operatorname {CP}_n({\mathbb {K}}))$. Similarly, if $\varvec{X}=\varvec{Y}\varvec{Y}^T\in \operatorname {CP}_n({\mathbb {K}})$, we deduce that $\operatorname {val}(\varvec{X})=\operatorname {val}(\varvec{Y})(\operatorname {val}(\varvec{Y})^T)$, showing that $\operatorname {val}(\operatorname {CP}_n({\mathbb {K}}))\subseteq \operatorname {CP}_n({\mathbb {T}})$. $\square$

The following result shows that the tropical analogue of the hierarchy (3) collapses.

#### Theorem 4.4

For all $$k\geqslant \max (n,\lfloor n^2/4\rfloor )$$,

$$\begin{aligned} \begin{aligned} \operatorname {val}(\operatorname {CP}_{n,k}({\mathbb {K}}))&= \operatorname {val}(\operatorname {CP}_n({\mathbb {K}})) = \operatorname {val}( \operatorname {CPSD}_{n,k}({\mathbb {K}}))\\&= \operatorname {val}( \operatorname {CPSD}_n({\mathbb {K}})) = \operatorname {val}(\operatorname {PSD}_n\cap {\mathbb {K}}_{\geqslant 0}^{n\times n}). \end{aligned} \end{aligned}$$

#### Proof

We use the following sequence of inclusions:

$$\begin{aligned} \begin{aligned} \operatorname {CP}_{n,k}({\mathbb {K}})&\subseteq \operatorname {CPSD}_{n,k}({\mathbb {K}}) \subseteq \operatorname {CPSD}_n({\mathbb {K}})\\&\subseteq \operatorname {PSD}_n\cap {\mathbb {K}}_{\geqslant 0}^{n\times n}\subseteq \{\varvec{X}:\varvec{X}_{ij}\geqslant 0,\ \varvec{X}_{ii}\varvec{X}_{jj}\geqslant \varvec{X}_{ij}^2\quad \forall i,j\}. \end{aligned} \end{aligned}$$

The last of these inclusions holds since the nonnegativity of all $2\times 2$ matrices is a necessary condition for a matrix to be positive semidefinite, and the rest of them follow from (3). Taking the valuation, we obtain

$$\begin{aligned} \operatorname {val}(\operatorname {CP}_n({\mathbb {K}}))&\subseteq \operatorname {val}(\operatorname {CPSD}_{n,k}({\mathbb {K}})) \subseteq \operatorname {val}(\operatorname {CPSD}_n({\mathbb {K}})) \subseteq \operatorname {val}(\operatorname {PSD}_n\cap {\mathbb {K}}_{\geqslant 0}^{n\times n})\\&\subseteq \operatorname {val}(\{\varvec{X}:\varvec{X}_{ij}\geqslant 0,\ \varvec{X}_{ii}\varvec{X}_{jj}\geqslant \varvec{X}_{ij}^2\quad \forall i,j\})\\&\subseteq \{X\in {\mathbb {T}}^{n\times n}:{X}_{ii}{X}_{jj}\geqslant {X}_{ij}^2\quad \forall i,j\}. \end{aligned}$$

By Theorem 4.3, the set $\{X\in {\mathbb {T}}^{n\times n}:{X}_{ii}{X}_{jj}\geqslant {X}_{ij}^2\quad \forall i,j\}$ is precisely $\operatorname {CP}_n({\mathbb {T}})$, which coincides with $\operatorname {CP}_{n,k}({\mathbb {T}})$ for all $k\geqslant \max (n,\lfloor n^2/4\rfloor )$. Moreover, by Proposition 4.2, $\operatorname {CP}_n({\mathbb {T}})$ coincides with $\operatorname {val}(\operatorname {CP}_n({\mathbb {K}}))$, hence, all the inclusions in the latter chain of inclusions must be equalities. $\square$

#### Remark 4.3

Note that given a matrix $\varvec{X}\in \operatorname {CP}_n({\mathbb {K}})$, the smallest integer *k* such that $\varvec{X}\in \operatorname {CP}_{n,k}({\mathbb {K}})$ is bounded by $$\left( {\begin{array}{c}n+1\\ 2\end{array}}\right) $$ (this result is proved by Hannah and Laffey [35] for matrices over ${\mathbb {R}}$, the same result is true over any real closed field, and in particular over ${\mathbb {K}}$). For the tropical completely positive cone there is a better bound $\max (n,\lfloor \frac{n^2}{4}\rfloor )$, proved in Cartwright and Chan [20], Theorem 4. This bound originated from the work of Drew, Johnson and Loewy [25] who conjectured that the CP-rank of any completely positive matrix over ${\mathbb {R}}$ is at most $\lfloor \frac{n^2}{4}\rfloor$. The bound was then shown to be generally false in the usual mathematics, in fact, Bomze, Schachinger and Ullrich [16] showed that $\operatorname {CP}_{n,k}({\mathbb {K}})\ne \operatorname {CP}_n({\mathbb {K}})$ for $k=n^2/2 +O(n^{3/2})$. However, the bound $\lfloor \frac{n^2}{4}\rfloor$ is true in tropical mathematics.

#### Remark 4.4

As pointed out in Prakash et al. [52], there is no general upper bound for the cpsd rank of a $n\times n$ cpsd matrix over a real closed field. It is shown there that this cpsd rank may be as high as $2^{\varOmega (\sqrt{n})}$. Hence, $\operatorname {CPSD}_{n,k}({\mathbb {K}})\ne \operatorname {CPSD}_n({\mathbb {K}})$ for $k=2^{O(\sqrt{n})}$, whereas Theorem 4.4 shows that the images of the two sets by the nonarchimedean valuation coincide.

### Tropical Copositive and Co-Completely Positive Semidefinite Matrices

Let us now observe that the *tropical copositive cone*, which we define as the tropical polar $\operatorname {CP}_n({\mathbb {T}})^{\circ }$ of the tropical completely positive cone, admits the same description as in the usual mathematics.

#### Lemma 4.3

We have

$$\begin{aligned} \operatorname {CP}_n({\mathbb {T}})^{\circ }=\{A\in ({\mathbb {S}}^{\vee })^{n\times n}\mid x^TAx\succcurlyeq {\mathbb {0}}\ \text { for all } x\in {\mathbb {T}}^n\}. \end{aligned}$$

#### Proof

For any $A\in \operatorname {CP}_n({\mathbb {T}})^{\circ }$ and any $x\in {\mathbb {T}}^n$ we have $\langle A,xx^T\rangle \succcurlyeq {\mathbb {0}}$, which is the same as $x^TAx\succcurlyeq {\mathbb {0}}$.

If $A\in ({\mathbb {S}}^{\vee })^{n\times n}$ satisfies this condition, then $\langle A,XX^T\rangle \succcurlyeq {\mathbb {0}}$ for any $X\in {\mathbb {T}}^{n\times k}$ since $XX^T=\bigoplus _{i=1}^k x^i (x^i)^T$ for the columns $x^i$ of *X*. Thus $A\in \operatorname {CP}_n({\mathbb {T}})^{\circ }.$ $\square$

We give a direct description of the same cone.

#### Theorem 4.5

We have $A\in \operatorname {CP}_n({\mathbb {T}})^{\circ }$ if and only if

$$\begin{aligned}  &   A_{ii} \succcurlyeq {\mathbb {0}}\quad \forall i\in [n],\\  &   \quad (A_{ij}^-)^2\preccurlyeq A_{ii}A_{jj}\quad \forall i,j\in [n]. \end{aligned}$$

#### Proof

Arguing as in the proof of Theorem 4.1, it suffices to establish the equivalence in the $2\times 2$ case, i.e., to show that, for all $a,b,c\in {\mathbb {S}}^\vee$,

$$\begin{aligned} ax_1^2 \oplus bx_1x_2 \oplus cx_2^2 \succcurlyeq {\mathbb {0}},\qquad \forall x_1,x_2\succcurlyeq {\mathbb {0}}\end{aligned}$$

holds if and only if

$$\begin{aligned} a,c\succcurlyeq {\mathbb {0}}\text { and } (b^-)^2 \preccurlyeq ac. \end{aligned}$$

The “Only if” part is established as in Lemma 4.1. Arguing as in the proof of this lemma, the only nontrivial case is when *a*, *c* are positive, and then, instead of $\mathbbm {1}\oplus \epsilon b/(\sqrt{a}\sqrt{c}) \succcurlyeq {\mathbb {0}}$ for all signs $\epsilon \in \{\mathbbm {1},\ominus \mathbbm {1}\}$, we only deduce that $\mathbbm {1}\oplus b/(\sqrt{a}\sqrt{c}) \succcurlyeq {\mathbb {0}}$. This gives $b^-\preccurlyeq \sqrt{a}\sqrt{c}$, i.e., $(b^-)^2\preccurlyeq ac$. The “If” part is established as in Lemma 4.1. $\square$

We now characterize $\operatorname {CP}_n({\mathbb {T}})^{\circ }$ in terms of the image of the classical cone of copositive matrices $\operatorname {CP}_n^{\circ }({\mathbb {K}})$ by the signed valuation.

#### Lemma 4.4

$$\operatorname {sval}(\operatorname {CP}_n^{\circ }({\mathbb {K}}))=(\operatorname {val}(\operatorname {CP}_n({\mathbb {K}}))^{\circ } =\operatorname {CP}_n({\mathbb {T}})^{\circ }$$.

#### Proof

By Proposition 4.2 we have $\operatorname {CP}_n({\mathbb {T}})=\operatorname {val}(\operatorname {CP}_n({\mathbb {K}}))$, so its tropical polar is $\operatorname {CP}_n({\mathbb {T}})^{\circ }$ is the same as $(\operatorname {val}(\operatorname {CP}_n({\mathbb {K}}))^{\circ }$. The rest of the claim then immediately follows from Theorem 3.4. $\square$

#### Theorem 4.6

For all $$k\geqslant \max (n,\lfloor n^2/4\rfloor )$$

$$\begin{aligned} \begin{aligned} \operatorname {sval}(\operatorname {PSD}_n({\mathbb {K}}) + {\mathbb {K}}_{\geqslant 0}^{n\times n})&=\operatorname {sval}( \operatorname {CPSD}_n^\circ ({\mathbb {K}}))=\operatorname {sval}(\operatorname {CPSD}_{n,k}^\circ ({\mathbb {K}}))\\&=\operatorname {sval}(\operatorname {CP}_{n,k}^{\circ }({\mathbb {K}})) =\operatorname {sval}(\operatorname {CP}_n^{\circ }({\mathbb {K}}))=\operatorname {CP}_n({\mathbb {T}})^{\circ }. \end{aligned} \end{aligned}$$

#### Proof

Theorem 4.4 implies

$$\begin{aligned} \begin{aligned} (\operatorname {val}(\operatorname {CP}_{n,k}({\mathbb {K}})))^{\circ }&= (\operatorname {val}(\operatorname {CP}_n({\mathbb {K}})))^{\circ } = (\operatorname {val}( \operatorname {CPSD}_{n,k}({\mathbb {K}})))^{\circ }\\&=(\operatorname {val}( \operatorname {CPSD}_n({\mathbb {K}})))^{\circ } = (\operatorname {val}(\operatorname {PSD}_n({\mathbb {K}}) \cap {\mathbb {K}}_{\geqslant 0}^{n\times n})^{\circ }. \end{aligned} \end{aligned}$$

for $k\geqslant \max (n,\lfloor n^2/4\rfloor )$. By Lemma 4.4, $(\operatorname {val}(\operatorname {CP}_n({\mathbb {K}})))^{\circ }=\operatorname {CP}_n({\mathbb {T}})^{\circ }$, so all of these polars coincide with $\operatorname {CP}_n({\mathbb {T}})^{\circ }$ and are stable under the operator $\operatorname {cl}\operatorname {int}(\cdot )$. Therefore we can apply Theorem 3.4, which transforms this chain of equalities to the one that is claimed. $\square$

#### Remark 4.5

The inclusion $\operatorname {sval}(\operatorname {PSD}_n)\subset \operatorname {sval}(\operatorname {PSD}_n+{\mathbb {K}}_{\geqslant 0}^{n\times n})$ is strict, since for example the matrix

$$\begin{aligned} A= \begin{pmatrix} 2 &  3 \\ 3&  2 \end{pmatrix} \end{aligned}$$

does not arise as the image of an element ${\textbf{A}}$ of $\operatorname {PSD}_n$ by the signed valuation (because the determinant of any such matrix ${\textbf{A}}$ should have signed valuation $\ominus 6$, and therefore, this determinant would be negative).

## Optimization in the Signed Tropical World

### Optimization Problems over the Symmetrized Tropical Semiring

We shall consider an optimization problem over ${\mathbb {S}}$ of the form:

$$\begin{aligned} \begin{aligned}&\inf \ f(x)\\&\quad \text { s.t. }\ x\in A\subseteq ({\mathbb {S}}^\vee )^n,\\&\quad f(x)\in {\mathbb {S}}^{\vee }, \end{aligned} \end{aligned}$$

where the $\inf$ is taken with respect to the $\succcurlyeq$ relation. It is important to require that $f(x)\in {\mathbb {S}}^{\vee }$, for otherwise $\succcurlyeq$ is not even an order relation (the transitivity breaks). The assumption that $A\subset ({\mathbb {S}}^{\vee })^n$ is relevant in applications, for as observed above, signed tropical numbers represent images of elements of ordered valued fields by the signed valuation.

#### Example 5.1

Consider the following problem

$$\begin{aligned} \begin{aligned}&\inf 4x^2\oplus 4x\oplus 0,\\&\quad \text { s.t. }\ x\in {\mathbb {S}}^{\vee }, 4x^2\oplus 4x\oplus 0\in {\mathbb {S}}^{\vee }. \end{aligned} \end{aligned}$$

For $f(x)=4x^2\oplus 4x\oplus 0$ and $x\in {\mathbb {S}}^{\vee }$ we have, in terms of the usual arithmetics, that

$$\begin{aligned} f(x)= {\left\{ \begin{array}{ll} 4+2|x|, &  \text { if } x\succcurlyeq 0 \text { or } x\prec \ominus 0,\\ 4+x, & \text { if } -4\preccurlyeq x\preccurlyeq 0,\\ 0, & \text { if } \ominus -4\prec x\preccurlyeq -4,\\ \ominus (4+|x|), & \text { if } \ominus 0\prec x\prec \ominus -4. \end{array}\right. } \end{aligned}$$

**Fig. 1:**
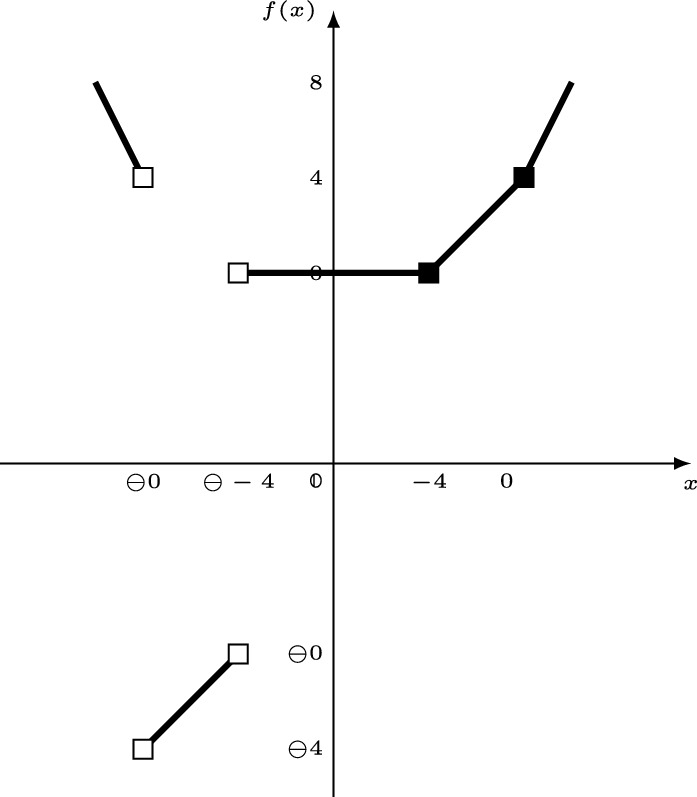
Polynomial *f*(*x*) over the symmetrized semiring

Note that when $x=\ominus -4$ and $\ominus 0$ then *f*(*x*) is balanced, so these values of *x* are excluded from optimization. The minimum of *f*(*x*) is $\ominus 4$ and it is obtained as *x* tends to $\ominus 0$ from the right, see Figure 1.

Let $f =\bigoplus _{k=0}^n a_k x^k \in {\mathbb {S}}^\vee [x]$ be a formal univariate polynomial of degree *n* (i.e., $a_n\ne {\mathbb {0}}$), with signed coefficients. We denote by $x\mapsto f(x)$ the associated polynomial function from ${\mathbb {S}}^\vee$ to ${\mathbb {S}}$, and let us also define the formal polynomial $|f|\in {\mathbb {T}}[x]$ by $|f|(x) = \bigoplus _{k=0}^n |a_k | x^k.$ The *roots* of *f* are defined as the points $x\in {\mathbb {S}}^\vee$ such that $f(x) \nabla {\mathbb {0}}$. The moduli of the roots of *f* are necessarily points of nondifferentiability of the map $x\mapsto |f|(x)$; however, not all nondifferentiability points yield roots (Baccelli et al. [13]). In fact, the notion of multiplicity of root in the setting of semirings or hyperfields with signs is a delicate one, see Baker and Lorscheid [14], Gunn [34].

We consider the optimization problem

15 $$\begin{aligned} \inf f(x) ;\qquad x\in {\mathbb {S}}^\vee , f(x) \in {\mathbb {S}}^\vee . \end{aligned}$$

Recall that ${\mathbb {S}}^\vee$ is equipped with the order topology. For all $\alpha \in {\mathbb {S}}^\vee$, we denote by $f(\alpha ^-)=\lim _{\beta \rightarrow \alpha , \beta \prec \alpha } f(\beta )$ and similarly $f(\alpha ^+)=\lim _{\beta \rightarrow \alpha , \beta \succ \alpha } f(\beta )$

The following proposition shows that optimization problems for univariate tropical polynomials admit an explicit solution.

#### Proposition 5.1

The following claims hold for (15):

(i) If the degree *n* is odd, the objective function of the optimization problem (15) is unbounded from below.
(ii) The same is true if the degree *n* is even, and if $a_n$ is negative.
(iii) If the degree *n* is even, if $a_n$ is positive, and if *f* has at least one root, then the value of the infimum is $\ominus |f|(|\alpha |)$, where $\alpha$ is any root of maximal modulus. Moreover, $\ominus |f|(|\alpha |) = f(\alpha ^-)$ if $\alpha$ is positive, and $\ominus |f|(|\alpha |) = f(\alpha ^+)$ if $\alpha$ is negative.
(iv) If the degree of *n* is even, if $a_n$ is positive, and if *f* has no root, then the value of the infimum is $f({\mathbb {0}})$.

#### Proof

We first observe that 1) the map $y\mapsto |f(y)|$ from ${\mathbb {T}}$ to ${\mathbb {S}}^\vee$ is increasing, 2) $f(x)=a_nx^n$ when

16 $$\begin{aligned} |x|\geqslant \bigoplus _{k=1}^n (|a_{n-k}|(|a_n|)^{-1})^{1/k}. \end{aligned}$$

We next prove all claims of the proposition.

(i),(ii): We observed that *f*(*x*) equals $a_nx^n$ for positive or negative $x\in {\mathbb {S}}^{\vee }$ satisfying (16). Let *n* be odd. If $a_n\succ {\mathbb {0}}$ then $f(x)=a_nx^n$ is unbounded from below as $x\rightarrow \bot$, and if $a_n\prec {\mathbb {0}}$ then the value $f(x)=a_nx^n$ is unbounded from below as $x\rightarrow \top$. If *n* is even and $a_n\prec {\mathbb {0}}$, then $f(x)=a_nx^n$ is unbounded from below as $x\rightarrow \bot$ and $x\rightarrow \top$.

(iii): In this case *f*(*x*), being equal to $a_nx^n$, stays positive for all positive or negative $x\in {\mathbb {S}}^{\vee }$ satisfying (16). If $\alpha$ is the root of *f* over ${\mathbb {S}}$ that has a maximal modulus and is positive, then *f*(*x*) remains positive for all $x\succ \alpha$, and *f*(*x*) changes sign at point $x=\alpha$. Considering any increasing sequence $\alpha _k\in {\mathbb {S}}^\vee$ converging to $\alpha$, we obtain that $\inf _k f(\alpha _k)= \ominus |f|(|\alpha |)$, and using the monotonicity property of $y\mapsto |f(y)|$, we get that the infimum of *f* over ${\mathbb {S}}^\vee$ is equal to $\ominus |f|(|\alpha |)$.

If $\alpha$ is the root of *f* over ${\mathbb {S}}$ that has a maximal modulus and is negative, then *f*(*x*) remains positive for all $x\prec \alpha$, and *f*(*x*) changes sign at point $x=\alpha$. Considering any decreasing sequence $\alpha _k\in {\mathbb {S}}^\vee$ converging to $\alpha$, we obtain that $\inf _k f(\alpha _k)= \ominus |f|(|\alpha |)$, and hence the infimum of *f* over ${\mathbb {S}}^\vee$ is equal to $\ominus |f|(|\alpha |)$ (using the monotonicity property of $y\mapsto |f(y)|$).

(iv): When *f* has no root, it follows from the monotonicity of $y\mapsto |f(y)|$ that the infimum is equal to $f({\mathbb {0}})$. $\square$

### Tropical Quadratic Programming

We now consider tropical quadratic programming problems. Given quadratic forms $f_i(x) = x^T A_i x \oplus b_i^T x \oplus c_i$ where $A_i\in ({\mathbb {S}}^\vee )^{n\times n}$, $b_i\in ({\mathbb {S}}^\vee )^{n}$, and $c_i\in {\mathbb {S}}^\vee$, for $i\in \{0,\dots ,m\}$,

17 $$\begin{aligned} \inf _{x\in ({\mathbb {S}}^\vee )^n} f_0(x) ; \qquad f_i(x)\preccurlyeq {\mathbb {0}}, \qquad i\in [m]. \end{aligned}$$

Recall that in our definition of an optimization problem, the infimum is taken over all *x* such that $f_0(x)$ is signed. The tropical quadratic feasibility problem consists in checking whether the inequalities $f_i(x)\preccurlyeq {\mathbb {0}}$ for all $i\in [m]$ hold for some $x\in ({\mathbb {S}}^\vee )^n$. We note that the two inequalities $f_i(x)\preccurlyeq {\mathbb {0}}$ and $f_i(x)\succcurlyeq {\mathbb {0}}$ are equivalent to $f_i(x)\nabla {\mathbb {0}}$, so “equalities” involving quadratic functions can be coded in this way. We also note that this formulation includes, as natural special case, the tropical linear programming problems studied by Butkovič [18], Ch. 10, Akian et al. [2], Gaubert et al. [31] and Allamigeon et al. [7]. Those problems, however, were posed over max-plus (tropical) semifield, i.e., with the decision variables confined to $({\mathbb {S}}^+)^n$ as opposed to $({\mathbb {S}}^{\vee })^n$ in Equation (17).

The following observation allows us to formulate tropical copositivity in terms of quadratic optimization.

#### Proposition 5.2

A matrix $A\in ({\mathbb {S}}^\vee )^{n\times n}$ is copositive if and only if the value of the quadratic optimization problem $\inf _{x\in ({\mathbb {S}}^\vee )^n}\{ x^T Ax ;\ x\succcurlyeq {\mathbb {0}}\}$ is ${\mathbb {0}}$.

#### Proof

Indeed, if *A* is copositive, we have by definition $x^T Ax \succcurlyeq {\mathbb {0}}$ for all $x\succcurlyeq {\mathbb {0}}$. Moreover, by taking $z={\mathbb {0}}$, we get $x^\top Ax={\mathbb {0}}\in {\mathbb {S}}^\vee$, showing that the above infimum is equal to ${\mathbb {0}}$. Conversely, suppose that *A* is not copositive. Then, there exists a vector $x\in ({\mathbb {S}}^\vee )^n$ such that $x^TAx\prec {\mathbb {0}}$ and $x^TAx$ is signed. Considering *ux* instead of *x*, with *u* positive, it follows that the value of the above infimum is bounded above by $u^2(x^TAx)$, for all such *u*, and so, the above infimum is equal to $\bot$. $\square$

Testing whether a matrix is copositive is a co-NP complete problem, see Dickinson and Gijben [24]. In contrast, it follows from Theorem 4.5 that checking whether a tropical matrix is copositive can be done in polynomial time. I.e., an important subclass of quadratic optimization problems which is NP-hard in the classical case becomes polynomial in the tropical case. However, the following result shows that, in general, tropical quadratic optimization remains NP-hard.

#### Theorem 5.1

The tropical quadratic feasibility problem is NP-hard.

#### Proof

Let 0 and 1 denote the usual zero and unit elements of ${\mathbb {R}}\subset {\mathbb {T}}\subset {\mathbb {S}}$, not to be confused with the ${\mathbb {0}}$ and $\mathbbm {1}$ elements of ${\mathbb {S}}$. The relations $s\in {\mathbb {S}}^\vee$ and $s^2\ominus 1 s\oplus 1\nabla {\mathbb {0}}$ are equivalent to $s\in \{0,1\}$ (to see this, note that $s^2\ominus 1 s\oplus 1= (s\ominus 0)(s\ominus 1)$. Then, if $x_i,y_i\in \{0,1\}$, the relation $x_iy_i \nabla 1$ for $i\in [m]$ is equivalent to “$x_i=0$ if and only if $y_i=1$”. In that way, the solutions of these relations encode *m* Boolean variables together with the negated variables. Consider now an instance of 3-SAT, in which the Boolean variables are $x_1,\dots ,x_m$. A clause $C_i$ can be written as $E \vee F \vee G$ where each term *E*, *F*, *G* is of the form $x_j$ or $\lnot x_j$ for some *j*. Let us interpret such a clause in ${\mathbb {S}}$, replacing $\lnot x_j$ by $y_j$, and replacing $\vee$ by $\oplus$. E.g., the validity of the clause $x_3\vee \lnot x_7\vee x_9$ is coded by the linear constraint $x_3 \oplus y_7 \oplus x_9 \nabla 1$. Recall also that a relation of the form $u\nabla v$ is equivalent to the conjunction of the two inequalities $u\preccurlyeq v$ and $u\succcurlyeq v$. In that way, we express an instance of 3-SAT as a collection of inequalities in the variables $x_1,\dots ,x_m,y_1,\dots ,y_m$. It follows that the tropical quadratic feasibility problem is NP-hard. $\square$

In view of the definition of signed tropically positive semidefinite matrices, it is natural to introduce also the class of tropical positive definite matrices:

$$\begin{aligned} \begin{aligned} \operatorname {PD}_n({\mathbb {S}})&=\{ A \in ({\mathbb {S}}^\vee )^{n\times n} \mid A_{ii}\succ {\mathbb {0}}, \forall i\in [n],\\&A_{ij}=A_{ji},\; A_{ij}^2 \prec A_{ii}A_{jj}, \forall i,j\in [n], i\ne j\}. \end{aligned} \end{aligned}$$

Let us now consider the following optimization problem with positive definite matrix *A*:

18 $$\begin{aligned} \inf _{x\in ({\mathbb {S}}^\vee )^n,\; x^TAx \oplus b^Tx\in {\mathbb {S}}^\vee } x^TAx \oplus b^Tx\in {\mathbb {S}}^\vee , \end{aligned}$$

We will also consider the following optimization problem:

19 $$\begin{aligned} \min _{{\textbf{x}}\in {\mathbb {K}}^n} {\textbf{x}}^T{\textbf{A}}{\textbf{x}}+{\textbf{b}}^T{\textbf{x}}, \end{aligned}$$

where $${\textbf{A}}={\textbf{A}}^T \in {\mathbb {K}}^{n\times n}$$ and $${\textbf{b}}\in {\mathbb {K}}^n$$.

We now define the *tropical comatrix* $\operatorname {com}A$ to be the matrix whose (*i*, *j*) entry equals $(\ominus \mathbbm {1})^{i+j} \det A(i,j)$ where *A*(*i*, *j*) is obtained by deleting row *i* and column *j* of *A*, and $\det$ denotes the determinant of a matrix with entries in ${\mathbb {S}}$, which is defined by the usual formula, see e.g. [1, 13] for background. Let $A=\operatorname {sval}({\textbf{A}})$ with ${\textbf{A}}\in {\mathbb {K}}^{n\times n}$. We say that the collection of entries of a set of matrices and vectors is generic if the vector of the moduli of the entries of this collection is generic, meaning that it belongs to the complement of a union of hyperplanes, see [10] for a similar condition. This holds in particular when it belongs to the complement of a union of tropical hypersurfaces, since the complement of a tropical hypersurface is necessarily the union of all the interiors of cells of maximal dimension of a polyhedral complex with full support [48]. If the collection of entries of *A* is generic, then, for all $i,j\in [n]$, there is only one term of maximal modulus in the expansion of $\det A(i,j)$ as a signed tropical sum of weights of $(n-1)!$ permutations, and it follows that $\operatorname {com}A =\operatorname {sval}\operatorname {com}{\textbf{A}}$ where $\operatorname {com}{\textbf{A}}$ denotes the usual comatrix of ${\textbf{A}}$.

#### Proposition 5.3

Suppose that $A\in ({\mathbb {S}}^\vee )^{n\times n}$ is positive definite, let $b\in ({\mathbb {S}}^\vee )^n$, and let ${\textbf{A}}={\textbf{A}}^T\in {\mathbb {K}}^{n\times n}$ and ${\textbf{b}}\in {\mathbb {K}}^n$ be such that $A=\operatorname {sval}{\textbf{A}}$ and $b=\operatorname {sval}{\textbf{b}}$. Then the following claims hold for (18) and (19):

(i) The optimal value of (18) is equal to $\ominus b^T \operatorname {diag}(A)^{-1} b$ and is obtained by considering a minimizing sequence converging to ${\bar{x}}:= \ominus \operatorname {diag}(A)^{-1} b$.
(ii) The optimal value of (18) coincides with the image by the signed nonarchimedean valuation of the optimal value of (19), equal to $-{\textbf{b}}^T{\textbf{A}}^{-1}{\textbf{b}}/4$.
(iii) If the collection of entries of $\{A,b\}$ is generic, then, the image by the signed nonarchimedean valuation of the unique optimal solution ${\textbf{x}}^*=-{\textbf{A}}^{-1}{\textbf{b}}/2$ of (19) is given by $x^*= \ominus (\det A)^{-1} (\operatorname {com}A)^T b$.

#### Proof

(i): Since we have, for all $x\in ({\mathbb {S}}^\vee )^n$ such that $x_i\ne {\mathbb {0}}$ and $x_j\ne {\mathbb {0}}$,

$$\begin{aligned} |x_iA_{ij}x_j|<|x_i|A_{ii}^{1/2}A_{jj}^{1/2}|x_j|\leqslant \max (x_i^2A_{ii},x_j^2A_{jj}), \end{aligned}$$

it is easy to see that $x^TAx=x^T\operatorname {diag}(A)x$ holds for all $x\in ({\mathbb {S}}^\vee )^n$. Using this we obtain that

$$\begin{aligned} x^TAx\oplus b^Tx=\bigoplus _{i=1}^n A_{ii}x_i^2\oplus b_ix_i\hspace{5.0pt}. \end{aligned}$$

Minimizing $a_{ii}x_i^2\oplus b_ix_i$ for each *i* we find (say, using Proposition 5.1) that since the only root is $\ominus A_{ii}^{-1}b_i$, the optimal value is attained by a minimizing sequence converging to this root, and the optimal value is $\ominus b_i A_{ii}^{-1}b_i$. The optimal value of (18) is then obtained by summing up these values, thus it is $\ominus b^T\operatorname {diag}(A)^{-1}b$, and it is attained by a minimizing sequence of vectors converging to $\ominus \operatorname {diag}(A)^{-1}b$.

(ii): We make the change of variables $x=Dx'$, where *D* is the diagonal matrix *D* with entries $D_{ii}=A_{ii}^{-1/2}$, which amounts to replacing *A* with $A'=DAD$ and *b* with $b'=Db$ in (18). We then have $A'_{ii}=\mathbbm {1}$ for all *i* and $A'_{ii}A'_{jj}=\mathbbm {1}> (A'_{ij})^2$ for all $i\ne j$, implying that $\det A' =\mathbbm {1}$. Then, we write $A'=I\ominus C$, where *C* is a matrix with diagonal entries equal to ${\mathbb {0}}$, and apply the last statement of Akian, Gaubert and Niv [4], Theorem 2.39, which entails that

20 $$\begin{aligned} (\operatorname {com}A')^T = C^*:=I \oplus C \oplus \dots \oplus C^{n-1} \hspace{5.0pt}. \end{aligned}$$

We also make the change of variables ${\textbf{x}}= {\textbf{D}} {\textbf{x}}'$, where ${\textbf{D}}\in {\mathbb {K}}^{n\times n}$ is the diagonal matrix with entries ${\textbf{D}}_{ii}=t^{D_{ii}}$, and set ${\textbf{A}}':={\textbf{D}}{\textbf{A}}{\textbf{D}}$ and ${\textbf{b}}':={\textbf{D}}{\textbf{b}}$. The value of the “lifted” problem (19) is given by

21 $$\begin{aligned} {\textbf{v}}= -\frac{\det ({\textbf{A}}')^{-1}}{4} {\textbf{b}}'^T (\operatorname {com}{\textbf{A}}')^T {\textbf{b}}' = -\frac{\det ({\textbf{A}}')^{-1}}{4} \sum _{i,j\in [n]} {\textbf{b}}'_i (\operatorname {com}{\textbf{A}}')_{ji} {\textbf{b}}'_j. \end{aligned}$$

Since $C_{ij}<\mathbbm {1}$ for all $i\ne j$, we deduce from (20) that, for $i\ne j$,

$$\begin{aligned} \operatorname {val}(\operatorname {com}{\textbf{A}}')^T_{ij} \leqslant |C_{ij}^*|<\mathbbm {1}. \end{aligned}$$

Moreover, $\operatorname {sval}(\operatorname {com}{\textbf{A}}')^T_{ii}=A'_{ii}=\mathbbm {1}$. Then, for $i\ne j$, $\operatorname {val}({\textbf{b}}'_i (\operatorname {com}{\textbf{A}}')_{ji}{\textbf{b}}'_j )< \operatorname {val}({{\textbf{b}}'}_i^2) \oplus \operatorname {val}({{\textbf{b}}'}_j^2) = \operatorname {val}({\textbf{b}}'_i (\operatorname {com}{\textbf{A}}')^T_{ii}{\textbf{b}}'_i) \oplus \operatorname {val}({\textbf{b}}'_j (\operatorname {com}{\textbf{A}}')^T_{jj}{\textbf{b}}'_j)$. Hence, in the sum in (21), only the diagonal terms, obtained when $i=j$, have a maximal valuation. We also observe that the sign of each of these terms is positive. Moreover, $\operatorname {sval}\det ({\textbf{A}}')=\mathbbm {1}$. It follows that $\operatorname {sval}{\textbf{v}} = \ominus b'^T (\det A')^{-1}(\operatorname {com}A')^T b'$. Using (20) and that $\det A' =\mathbbm {1}$ this becomes

$$\begin{aligned} \ominus \bigoplus _{i,j\in [n]} b'_{i}C^*_{ij}b'_j= \ominus \bigoplus _{i\in [n]} b_iA_{ii}^{-1}b_i =\ominus b^T \operatorname {diag}(A)^{-1}b, \end{aligned}$$

as claimed.

(iii): If the collection of entries of *A* is generic, we noted above that the matrix $\operatorname {com}A = \operatorname {sval}\operatorname {com}{\textbf{A}}$ is signed. If in addition the collection of entries of $\{A,b\}$ is generic, then the entries of ${\operatorname {com}A}^Tb$ are also signed and ${\operatorname {com}A}^Tb = \operatorname {sval}{\operatorname {com}{\textbf{A}}}^T {\textbf{b}}$. We finally claim that, since *A* is positive definite, we have

22 $$\begin{aligned} \det A = \bigodot _{i\in [n]}A_{ii} =\operatorname {sval}\det {\textbf{A}}\hspace{5.0pt}. \end{aligned}$$

Indeed, after making the change of variables of (ii), we may assume that $A_{ii}=\mathbbm {1}$ for all *i*, with $A_{ii}A_{jj}=\mathbbm {1}> A_{ij}^2$. Considering the expansion of $\det A$, we see that the identity permutation yields the unique term with maximal modulus, from which (22) readily follows. These facts imply that $x^*$ is the image by signed valuation of ${\textbf{x}}^*$. $\square$

#### Remark 5.1

The valuation of the optimal solution $x^*=\operatorname {sval}{\textbf{x}}^*$ may differ from the vector ${\bar{x}} = \ominus \operatorname {diag}(A)^{-1} b$ defined in (i). Indeed, consider

$$\begin{aligned} A = \left( \begin{array}{cc} 0 &  \ominus (-1)\\ \ominus (-1) &  0 \end{array}\right) , \quad {\textbf{A}}= \left( \begin{array}{cc} 1 &  - t^{-1}\\ - t^{-1} &  1 \end{array}\right) , \quad b = \left( \begin{array}{c} 0\\ \theta \end{array}\right) , \quad {\textbf{b}}= \left( \begin{array}{c} 1\\ t^{\theta } \end{array}\right) , \end{aligned}$$

with $\theta \in {\mathbb {R}}\subset {\mathbb {S}}^\vee$, so that $A=\operatorname {sval}{\textbf{A}}$ and $b=\operatorname {sval}{\textbf{b}}$. We have ${\textbf{x}}^*= -(1-t^{-2})^{-1}(1 + t^{-1} t^{\theta }, t^\theta + t^{-1})^T$, $x^*=\ominus (\mathbbm {1}\oplus (-1 +\theta ), \theta \oplus (-1))^T$, whereas ${\bar{x}}=\ominus (0,\theta )^T$. We observe that for $\theta >1$, $x^*=\ominus ((-1+\theta ), \theta )^T \ne {\bar{x}}$.

## Concluding Remarks

In this paper we have characterized the polars of subsets *A* of ${\mathbb {T}}^n$ in terms of signed elimination cones (cones of signed vectors, stable under a special addition). Upon introducing and characterizing the tropical analogues of positive semidefinite matrices, completely positive semidefinite matrices and copositive matrices we showed that both the hierarchy of classical matrix cones and the hierarchy of their polars collapse under signed tropicalization. We also studied some optimization problems over symmetrized tropical semiring. Let us now also mention the following two future research directions arising from this research.

Firstly, instead of defining the tropical polar of a subset *A* of ${\mathbb {T}}^n$ we may more generally consider a subset *A* of $({\mathbb {S}}^\vee )^n$ and still define its signed polar as in (5). This leads to a larger class of signed polars, and it would be interesting to extend the present results to this class.

Secondly, in Section 5, we only made some “first steps” in optimization over signed tropical numbers, by considering the tropical quadratic feasibility problem and the unconstrained optimization of tropical polynomials of one variable and tropical quadratic functions. In particular, we showed that the membership in the tropical copositive cone can be checked in polynomial time, whereas the analogous problem in the classical world is NP-hard. Further relations between tropical and classical optimization and applications of such relations are to be explored in the future.

We also note that the nature of this work is theoretical and our conclusions and findings are not relevant to any data set.
